# Differences in Gastric Carcinoma Microenvironment Stratify According to EBV Infection Intensity: Implications for Possible Immune Adjuvant Therapy

**DOI:** 10.1371/journal.ppat.1003341

**Published:** 2013-05-09

**Authors:** Michael J. Strong, Guorong Xu, Joseph Coco, Carl Baribault, Dass S. Vinay, Michelle R. Lacey, Amy L. Strong, Teresa A. Lehman, Michael B. Seddon, Zhen Lin, Monica Concha, Melody Baddoo, MaryBeth Ferris, Kenneth F. Swan, Deborah E. Sullivan, Matthew E. Burow, Christopher M. Taylor, Erik K. Flemington

**Affiliations:** 1 Department of Pathology, Tulane University, New Orleans, Louisiana, United States of America; 2 Tulane Cancer Center, New Orleans, Louisiana, United States of America; 3 Department of Computer Science, University of New Orleans, New Orleans, Louisiana, United States of America; 4 Department of Mathematics, Tulane University, New Orleans, Louisiana, United States of America; 5 Department of Medicine, Section of Clinical Immunology, Allergy, and Rheumatology, Tulane University, New Orleans, Louisiana, United States of America; 6 Tulane Center for Stem Cell Research and Regenerative Medicine, New Orleans, Louisiana, United States of America; 7 BioServe Biotechnologies, Ltd., Beltsville, Maryland, United States of America; 8 Department of Microbiology & Immunology, Tulane University, New Orleans, Louisiana, United States of America; 9 Department of Obstetrics and Gynecology, Tulane University, New Orleans, Louisiana, United States of America; 10 Department of Medicine, Section of Hematology and Medical Oncology, Tulane University, New Orleans, Louisiana, United States of America; 11 Department of Microbiology, Immunology & Parasitology, Louisiana State University School of Medicine, New Orleans, Louisiana, United States of America; 12 Research Institute for Children, Children's Hospital, New Orleans, Louisiana, United States of America; Wistar Institute, United States of America

## Abstract

Epstein-Barr virus (EBV) is associated with roughly 10% of gastric carcinomas worldwide (EBVaGC). Although previous investigations provide a strong link between EBV and gastric carcinomas, these studies were performed using selected EBV gene probes. Using a cohort of gastric carcinoma RNA-seq data sets from The Cancer Genome Atlas (TCGA), we performed a quantitative and global assessment of EBV gene expression in gastric carcinomas and assessed EBV associated cellular pathway alterations. EBV transcripts were detected in 17% of samples but these samples varied significantly in EBV coverage depth. In four samples with the highest EBV coverage (hiEBVaGC – high EBV associated gastric carcinoma), transcripts from the BamHI A region comprised the majority of EBV reads. Expression of LMP2, and to a lesser extent, LMP1 were also observed as was evidence of abortive lytic replication. Analysis of cellular gene expression indicated significant immune cell infiltration and a predominant IFNG response in samples expressing high levels of EBV transcripts relative to samples expressing low or no EBV transcripts. Despite the apparent immune cell infiltration, high levels of the cytotoxic T-cell (CTL) and natural killer (NK) cell inhibitor, IDO1, was observed in the hiEBVaGCs samples suggesting an active tolerance inducing pathway in this subgroup. These results were confirmed in a separate cohort of 21 Vietnamese gastric carcinoma samples using qRT-PCR and on tissue samples using in situ hybridization and immunohistochemistry. Lastly, a panel of tumor suppressors and candidate oncogenes were expressed at lower levels in hiEBVaGC versus EBV-low and EBV-negative gastric cancers suggesting the direct regulation of tumor pathways by EBV.

## Author Summary

Epstein-Barr virus (EBV) is detected in roughly 10% of gastric carcinoma (GC) cases worldwide. Despite a strong link between EBV and gastric carcinoma, the contribution of EBV to the tumor environment in EBV associated gastric carcinoma is unclear. We performed a global assessment of EBV and host cell gene expression in gastric carcinoma tumors from 71 patients to link EBV genes (and expression intensities) to cell and microenvironmental changes. In addition to the finding that EBV is associated with down-regulated tumor regulatory genes, this study revealed that samples with high levels of EBV gene expression (hiEBVaGCs) displayed elevated immune cell infiltration with high interferon-gamma (IFNG) expression compared to samples with low or no EBV gene expression. Despite this evidence of increased immune posturing, hiEBVaGC samples also showed elevated expression of the potent immune cell inhibitor, IDO1. This finding may partly explain the persistence of these virus associated tumors in the face of local immune cell concentration. Importantly, the small molecule IDO inhibitor, 1MT (1-methyl Tryptophan), has been shown to reverse the tolerance inducing effects of IDO1 in other tumors. We propose that stratification of gastric carcinomas into EBV-negative, EBV-low and EBV-high may provide indicator value for the use of IDO1 inhibitors as adjuvant therapies against hiEBVaGCs.

## Introduction

Epstein-Barr virus (EBV) is a herpes virus that infects most humans by adulthood. EBV is associated with several human malignancies, including malignancies of epithelial origin. The first report showing EBV's association with lymphoepithelioma-like carcinomas of the stomach was in 1990 by Burke and colleagues using polymerase chain reaction (PCR) [1]. Since that time, several studies have investigated the association between EBV and gastric carcinomas using a variety of methods (PCR, Southern blotting, and in situ hybridization (ISH)). In 1992, Shibata and Weiss reported EBV infection in 16% of gastric adenocarcinomas using PCR primers to the EBNA 1 gene and by ISH using probes against the EBV encoded small RNAs, EBERs [2]. Another report from Japan detected EBV in 6.9% of gastric carcinoma cases using EBER ISH [3]. Attributed to regional/country differences, the highest incidence of EBV-associated gastric carcinoma (EBVaGC) (16%) has been reported from the United States [2] while the lowest incidence (1.3%) is from Papua New Guinea [4]. Despite these landmark studies showing the association between gastric carcinomas and EBV, the mechanisms of EBV pathogenesis in gastric carcinoma are unclear.

Previous studies have shown the sensitivity of high throughout sequencing for detecting infectious agents [5], [6], [7] and for the new discovery of exogenous agents associating with human cancer [6], [8]. Merkel cell virus has been linked to Merkel carcinoma [8] and Fusobacterium has recently been associated with colorectal carcinoma [6]. In line with other reported methods for investigating pathogen associations in human cancers, we have previously developed a computational pipeline for the identification of exogenous sequences in RNA-seq data called PARSES [9]. Using PARSES, we examined two B-cell lines, Akata and JY, which are commonly used as model systems for EBV studies. Analysis of these cell lines revealed the presence of EBV in both cell lines as expected, but it also revealed the presence of the murine leukemia virus, MuLV in the JY but not Akata cell lines [7].

We have improved PARSES to include the utilization of parallel computing either on a local cluster or large-scale clusters, and we have included features that allow the user to simultaneously analyze the human cellular genes in addition to pathogen discovery (recently coined as ‘dual RNA-seq’ by Westermann and colleagues [10]). Here we utilized this pipeline, RNA CoMPASS (RNA comprehensive multi-processor analysis system for sequencing, Xu et al., unpublished), for the detection of viral pathogens in clinical tumor samples by analyzing a cohort of gastric carcinomas generated by the Cancer Genome Atlas initiative (SRA035410). EBV was detected in 12 out of 71 gastric carcinoma samples and the depth of coverage was sufficient to assess overall transcriptome structure in four cases. To our knowledge, this is the first study to globally assess both the EBV and host transcriptomes in gastric carcinomas using RNA-seq (although a recent paper has shed light onto this EBV specific host cell changes using a real time RT-PCR approach [11]). Our analysis led to insights into viral-host interactions and mechanisms through which EBV alters its local tumor environment. Further, this analysis revealed significant differences in the degree of host responses depending on the level of EBV gene expression. This raises the idea that the magnitude may be a more important clinical indicator than the simple detection of EBV in the selection of therapeutic regimens and the prediction of therapeutic responses in gastric carcinomas.

## Results

### Detection of EBV in gastric adenocarcinoma samples using RNA CoMPASS

**Figure 1:**
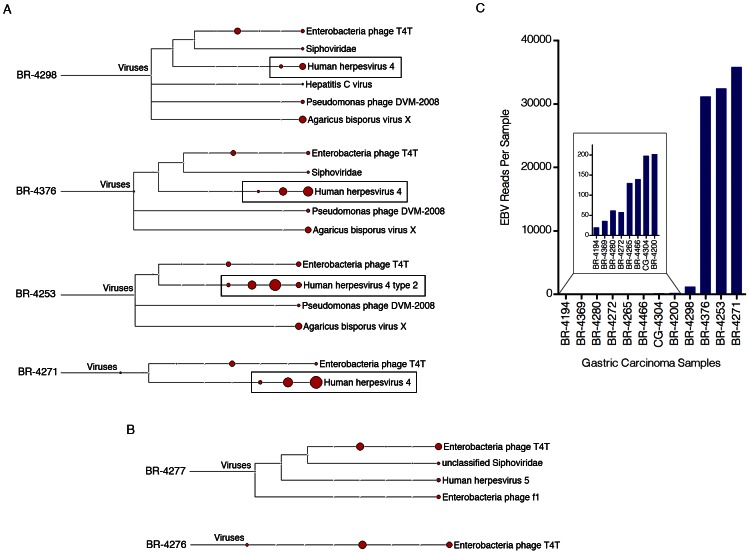
Detection of EBV in gastric carcinoma samples. Four gigabytes of deduplicated RNA-seq read data from each of the seventy-one gastric carcinoma samples were analyzed using RNA CoMPASS. The virome branch of the taxonomy trees for the four samples with the highest number of EBV reads (A) and two EBV-negative samples (B) were generated using the metagenome analysis tool, MEGAN 4. (C) For a more in depth analysis of EBV reads, the combined sequence read files for each sample were aligned to the EBV genome and the hg19 human genome assembly using the genome aligner, Novoalign. Of the EBV-positive samples, four samples were identified as having high numbers of EBV reads while eight were found to have low but detectable numbers of EBV reads (see Figure S2 for plot of EBV reads per 1,000,000 human mapped reads).

RNA-seq data from The Cancer Genome Atlas (TCGA) gastric adenocarcinoma cohort (SRA035410) was first analyzed using RNA CoMPASS (Figure S1 and Xu et al., unpublished) to assess the virome for each of the 71 data sets. This initial screening was performed using a single lane of sequencing data from each patient. Most samples contained relatively low numbers of reads matching non-human viral sources (e.g. enterobacteria phage T4T) that possibly represent environmental contamination (Figure 1A–B). Of the known human viruses detected, one sample (BR-4298, Figure 1A) contained 6 reads attributed to Hepatitis C virus. Further inspection of these reads showed high homology to the human immunoglobulin light chain variable region (Table S1). These reads likely represent human sequences rather than reads derived from Hepatitis C virus. Twelve samples showed evidence of human cytomegalovirus (HCMV) with read numbers ranging from 5 to 132. Individual BLASTing of selected HCMV reads showed high homology to HCMV genomes but not to human sequences indicating that these are bona fide HCMV derived reads. The relatively low numbers of HCMV reads in these samples (relative to the numbers of EBV in some samples, see below) suggests that these reads are derived from a low number of HCMV infected cells or that the virus is not expressing substantial numbers of polyadenylated RNAs in these tumor samples.

EBV was detected in 12 out of the 71 (17%) gastric carcinoma cases with varying levels of reads. To further analyze the EBV-associated gastric carcinoma (EBVaGC) samples, the two lanes of sequence per sample were combined to attain greater sequencing depth. These sequence files were aligned against a modified EBV B95-8 genome that contains Raji genome sequences inserted into a deleted region of the B95-8 genome (Genbank accession number AJ507799) plus the hg19 assembly of the human genome. Alignments were carried out using Novoalign V2.07.18 [-o SAM, paired-end, default options]. Based on the assembly to the human genome, sample quality and throughput was found to be consistent across all samples with the numbers of human mapped reads ranging from 128 to 159 million. Eight of the 12 EBV positive samples were found to have less than 200 reads per sample (inset of Figure 1C), three were found to contain more than 30,000 reads and one sample was found to contain 1,194 reads (Figure 1C). We tentatively considered the 8 cases with less than 200 reads to represent nominal infections similar to that observed with CMV (above). The 4 samples with higher read numbers, BR-4253, BR-4271, BR-4376, and BR-4298, were taken for more in depth transcriptome analysis.

Notably, while three of the four EBV positive samples with high numbers of EBV reads were classified as the more common Type I strain of EBV, one of these samples, BR-4253, was classified as the type II strain (Figure 1A). Since the strain defining regions of EBV, EBNA2 and EBNA3A/3B/3C [12] are largely not expressed in EBVaGC, we were concerned that the reads from sample BR-4253 could be misclassified as type II. We analyzed a few of the reads defined by MEGAN as type II from sample BR-4253 using manual BLAST and the majority of reads aligned to both type I (B95-8/Raji) and type II strains (AG876 (Genbank accession number DQ279927)) with some of these showing better homology to AG876 (data not shown). Despite this, the small number of reads derived from the EBNA2 and EBNA3A/3B/3C loci were more homologous to the type I than the type II strain. Therefore, this sample was likely misclassified as the type II strain because of greater similarity to the AG876 genome at highly expressed regions outside of the EBNA2 and EBNA3A/3B/3C loci.

### EBV gene expression in gastric carcinomas

**Figure 2:**
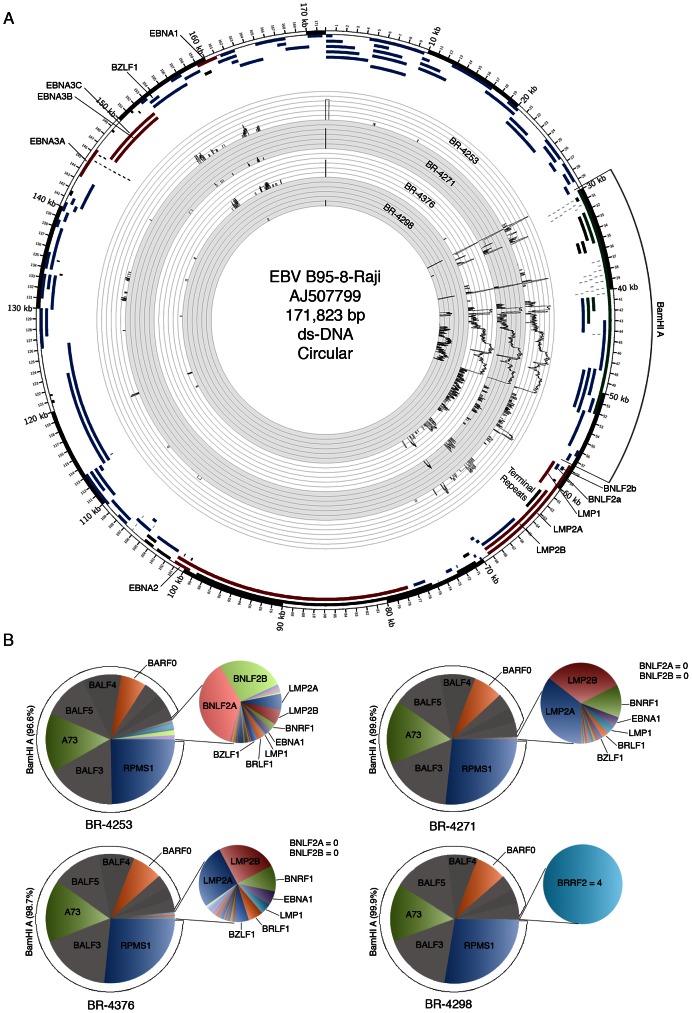
Genome wide analysis of EBV gene expression. (A) An annotated Circos plot depicting EBV read coverage across the EBV genome. Coverage graphs display the number of reads mapping to each nucleotide position of the genome and are depicted in log scale. Expandable log and non-log plots are provided in Figures S3 and S4. Note that alignments were performed using a genome that was split between the BBLF2/3 and the BGLF3.5 lytic genes rather than at the terminal repeats to accommodate coverage of splice junctions for the latency membrane protein, LMP2. The natural termini of the linear genome, the terminal repeats, are shown in the lower right quadrant of the graph. Coverage data is plotted relative to the modified B95-8 genome containing Raji genome sequences (Genbank accession number AJ507799). Blue features represent lytic genes, red features represent latency genes, green features represent potential non-coding genes, aquamarine features represent microRNAs, and black features represent non-gene features (repeat regions and origins of replication, for example). (B) Pie charts displaying read counts across EBV gene features. Because the BNLF2a/b region is contained within the LMP1 gene, total LMP1 read counts were inferred by determining the counts within the unique LMP1 sequences, multiplying by the total length of LMP1, and dividing by the length of the unique region. BNLF2a/b counts were calculated by determining the number of reads within the BNLF2a/b locus and subtracting the inferred number of LMP1 reads derived from within the BNLF2a/b coordinates (i.e. number of LMP1 reads within the unique region times the length of the overlap region divided by the length of the unique region). Leftward oriented genes within the BamHI A region are shown in grey. This representation indicates uncertainty due to the finding of primarily rightward transcription across these genes in the gastric carcinoma cell line SNU-719 using directional sequencing methods (see below).

EBV transcript quantification and genome coverage information was generated for samples, BR-4253, BR-4271, BR-4376, and BR-4298 using the transcriptome analysis software, SAMMate (note that the sequencing libraries were generated from polyA selected RNA which precludes the sequencing of EBER genes) [13]. Genome coverage information was first visualized by displaying the number of reads across each genomic position in the Circos plot shown in Figure 2A. Because of disparate coverage intensities, the Circos graph in Figure 2A is plotted in log scale to allow simultaneous visualization of the less abundantly expressed regions of the genome (expandable non-log and log plots are provided in Figures S3 and S4). Notably, coverage across the BamHI A region was high relative to other parts of the genome with greater than 96% of total reads corresponding to the BamHI A region in each case (Figure 2B).

Evidence for transcription of the essential episomal replication factor, EBNA1 is observed in samples BR-4253, BR-4271 and BR-4376 (Figure 2A (upper left region of the figure) and Figure 2B). No EBNA1 reads were detected in BR-4298 most likely owing to the significantly lower read numbers in this sample (Figures 2A–B).

**Figure 3:**
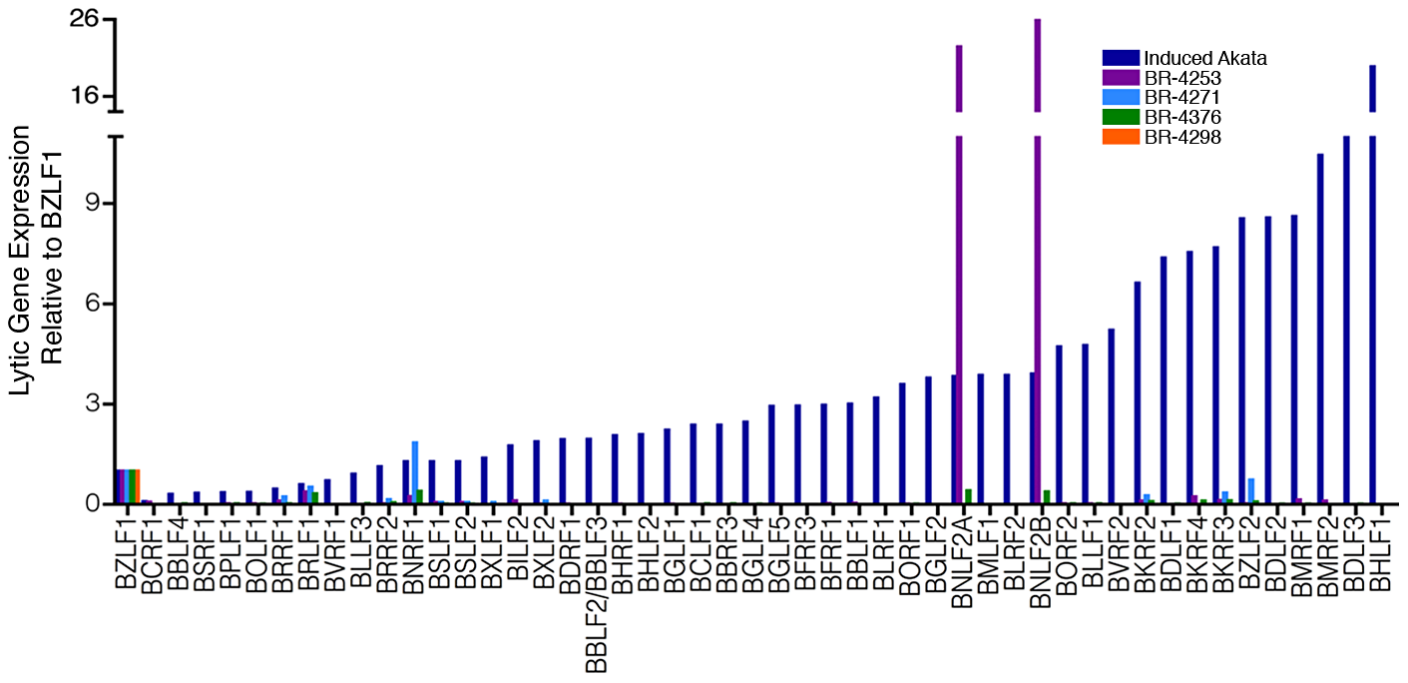
Abortive lytic gene expression. EBV lytic gene expression in EBVaGC samples. Lytic gene expression relative to BZLF1 represents RPKMs (reads per kilobase of exon model per million mapped reads) for each indicated gene divided by the RPKMs of BZLF1 for the respective biological sample. For reference to a productive replication setting, samples were compared to the lytic gene expression profile in reactivated Akata cells.

Evidence for transcription of the immediate early genes, BZLF1 and BRLF1, is similarly seen in BR-4253, BR-4271 and BR-4376 but not in BR-4298 (again, possibly due to the low overall read numbers). Despite the detection of BZLF1 and BRLF1 reads in samples BR-4253, BR-4271 and BR-4376, there is a remarkable absence of reads for most other downstream lytic genes in these samples. In Figure 3, we plotted the ratio of lytic gene transcripts (*sans* lytic genes in the BamHI A region) relative to the level of BZLF1 RNAs in BR-4253, BR-4271 and BR-4376 and compared this to the corresponding relative levels of these gene transcripts in reactivating Akata cells [14]. This comparison indicates that while the BZLF1 and BRLF1 immediate early genes are expressed in these tumors, there is a clear lack of lytic cycle progression; reflecting abortive lytic replication in this *in vivo* setting.

**Figure 4:**
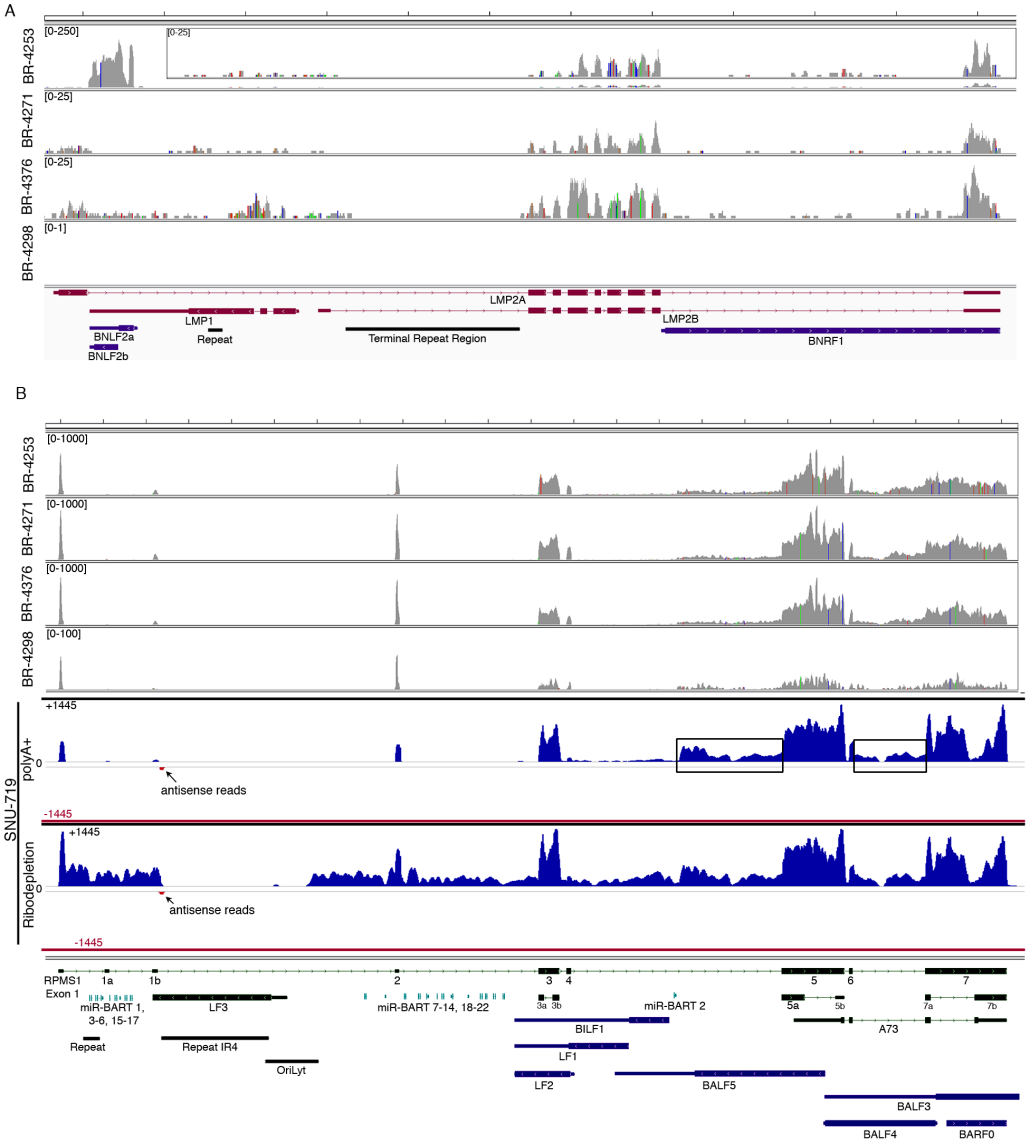
EBV gene expression analysis. Detailed read coverage data for the LMP2a, LMP1, and BNLF2a/b genes (A) and the RPMS1/BamHI A regions (B) of the EBV genome. Data was displayed using the Integrative Genomics Viewer (IGV) using the modified B95-8 genome containing Raji genome sequences (Genbank accession number AJ507799). The y axis represents the number of reads at each nucleotide position of the genome. Blue features represent lytic genes, red features represent latency genes, green features represent potential non-coding genes, aquamarine features represent microRNAs, and black features represent non-gene features (repeat regions and origins of replication, for example). In panel (A), coverage graphs for BR-4253 is scaled to a maximum read level of 250 reads (the BR-4253 inset displays the data with a max read level of 25), the BR-4271 and BR-4376 graphs are scaled to a max read level of 25, while the max read level for BR-4298 is 1. For coverage across the RPMS1/BamHI A region (B), BR-4253, BR-4271, and BR-4376 are scaled to 1,000 reads, while BR-4298 is scaled to 100. Strand specific sequencing from SNU-719 cells of the RPMS1/BamHI A region is also displayed. The top 2 tracks are from poly(A) selected RNA and the bottom 2 tracks are from Ribo-Zero depleted RNA. The read coverage for the sense strand is displayed in blue with positive values while the antisense strand is displayed in red with negative values. The scale is + or −1,445 reads for the sense and antisense strands.

Consistent with previous reports of LMP2 expression in gastric carcinomas [15], [16], we similarly see evidence of LMP2 transcription in samples BR-4253, BR-4271 and BR-4376 (Figures 2A–B and Figure 4A). LMP1 has been previously reported to be expressed at low levels or to be not expressed in gastric carcinomas [17], [18], [19]. We similarly find low albeit detectable levels of LMP1 in BR-4253, BR-4271 and BR-4376 (Figures 2B and 4A). Strikingly, however, sample BR-4253 has a very high number of reads corresponding to the early BNLF2A/B locus, which overlaps the LMP1 3′ untranslated region (Figures 2B, 3 and 4A). No BNLF2A/B reads are detected in BR-4271, BR-4376, and BR-4298 (Figure 2B) suggesting that this is unique to BR-4253. The high expression level of the early BNLF2A/B genes in BR-4253 is surprising because it occurs in the absence of most other early genes. This suggests the possibility that BNLF2A/B is expressed in this patient through an alternative mechanism possibly mediated through a viral genetic alteration.

### Analysis of the highly expressed BamHI A region

The most actively polyA transcribed region of the EBV genome, the BamHI A region (Figures 2A–B), shows excellent coverage across most of the RPMS1/A73 exons with apparent additional coverage observed for the regions spanning the leftward transcribed genes, BALF5, BALF3, and BALF4 (Figure 4B). Coverage across these leftward genes is unexpected because they are thought to be lytic genes and not expressed during latency. We therefore performed directional sequencing of a naturally occurring EBV positive gastric carcinoma cell line, SNU-719, to allow us to determine the orientation of transcripts across this region. EBV read coverage for SNU-719 was remarkably similar to that observed for the tumor specimens (Figure 4B). Outside of a small blip of leftward transcription noted near the RPMS1 exon 1b, there is little leftward transcription across this region. This indicates that the transcription observed across this region in the tumor specimens are likely rightward oriented and to a large extent related to RPMS1 and/or A73 but not BALF5, BALF3, BALF4, BILF1, LF1, or LF2.

Also notable in Figure 4B is rightward coverage across the introns between exons 4 and 5 and exons 6 and 7 of the RPMS1 gene (boxed regions in SNU-719 tracks). This coverage likely does not represent intron fragments generated after transcript splicing because this coverage is observed in sequencing libraries generated from polyA selected RNA (upper SNU-719 tracks). In contrast, there is no coverage of the first 4 RPMS1 introns on the polyA track whereas there is substantial coverage across these regions when ribo-depleted RNA was used for sequencing (Figure 4B). Therefore, the rightward coverage between exons 4 and 5 and between exons 6 and 7 likely represent bona fide previously unannotated rightward exons/transcripts. The read coverage between exons 6 and 7 may arise from mature RPMS1 isoforms that retain this intron (forming a unique RPMS1 isoform). The coverage between exons 4 and 5 starts near the middle of this intron suggesting that this is a site of transcription initiation or a that it is a splice acceptor site. Since splice mapping (see below) did not identify candidate splicing events near the beginning of this intron coverage, it is possible that this coverage arises from transcription initiation from an unknown upstream promoter.

**Figure 5:**
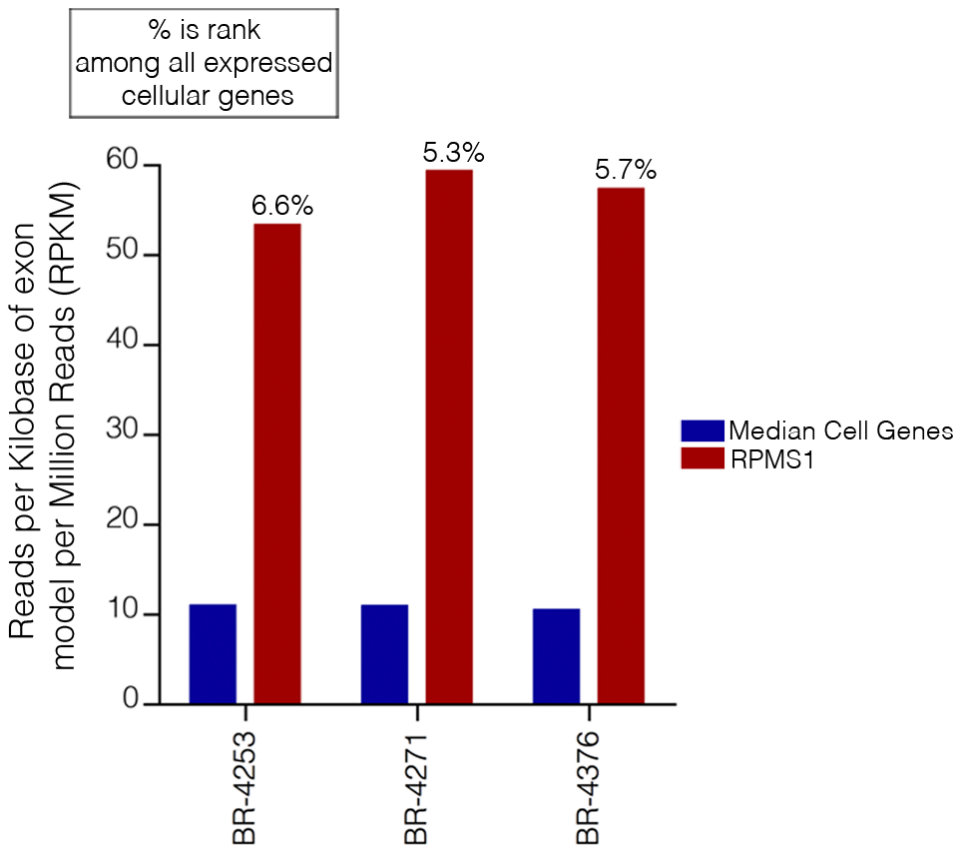
EBV transcripts from RPMS1 are among the highest expressed genes in EBVaGCs. RPKM values calculated using reads across all RPMS1 exons are shown with respect to the median expression of all expressed cellular genes (expressed genes defined as cellular genes with greater than 1 RPKM). The percentage values above each RPMS1 bar represents the rank of RPMS1 expression in the respective sample among all expressed cellular genes in that sample.

As mentioned above, more than 96% of all EBV reads align to the BamHI A region. Further, RPMS1 exon coverage ranks within the top seven percent of expressed cellular genes in samples BR-4253, BR-4271, and BR-4376 with expression that is more than five times the median cellular gene expression level (Figure 5). We conclude that not only is expression of this region high relative to other EBV encoded genes, but the expression is also high relative to cellular genes. In contrast, it is notable that the LF3 gene which is within the BamHI A locus and which has been found to be expressed at very high levels in other systems [20], shows no evidence of expression in these *in vivo* gastric carcinoma tumor datasets.

**Figure 6:**
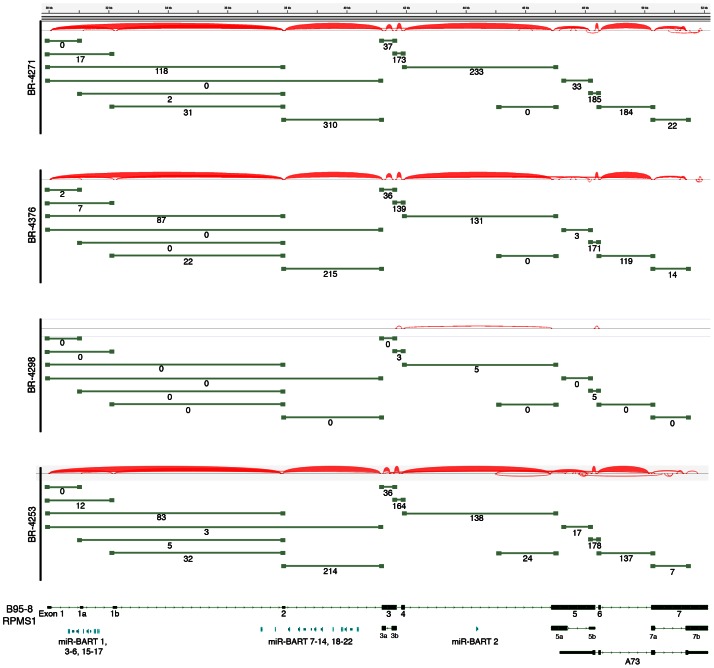
Alternative splicing in the EBV BamH1 A region in EBVaGCs. RNA-seq data from BR-4271, BR-4376, and BR-4298 and BR-4253 was analyzed using the TopHat aligner to obtain splice junction information. Samples with the type I strain of EBV, BR-4271, BR-4376, BR-4298, and BR-4253 were aligned to the type I genome, B95-8/Raji (Genbank accession number AJ507799). Junctions were visualized using Integrative Genomic Viewer (IGV) [88]. Thickness of red junction features correlates with the number of reads for the respective junction. The number of junction spanning reads for each junction is indicated below each olive green junction feature.

To assess splicing events in this region, alignments were performed using the junction mapper, TopHat [21]. Consideration of the most abundant splice junction reads indicates the predominance of sequential splicing from exons 1-2-3-4-5-6-7 (Figure 6). Nevertheless, there is significant evidence of intra-exonal splicing at exons 3 (3a to 3b), 5 (5a to 5b), and 7 (7a to 7b) (Figure 6). Although splicing from exons 1 to 2 is the most predominant 5′ region splicing order, there is also good evidence of alternative splicing to exon 1a (i.e. splicing of exon 1 to exon 1a to exon 2) (Figure 6). In samples BR-4253 (Figure 6) and SNU-719 (data not shown), we also noted evidence of splicing initiating from the middle of the newly identified coverage in the intron between exons 4 to 5 to the start of exon 5. This indicates additional complexity in this new region whereby some of these transcripts splice to exon 5 while some read through to exon 5.

### Differential cellular gene expression patterns in EBV associated and EBV low/negative gastric adenocarcinomas

EBV likely contributes to gastric carcinoma through the subversion of at least some of the oncogenic pathways required for the development of gastric carcinoma. However, the way that EBV subverts these pathways is likely distinct from the mechanism of pathway disruption in the absence of EBV (e.g. through genetic alterations). Since cellular gene expression is typically responsive to altered signaling mechanisms, differences in gene expression profiles can be used to not only classify cell populations but also infer upstream signaling events within certain cell populations.

**Figure 7:**
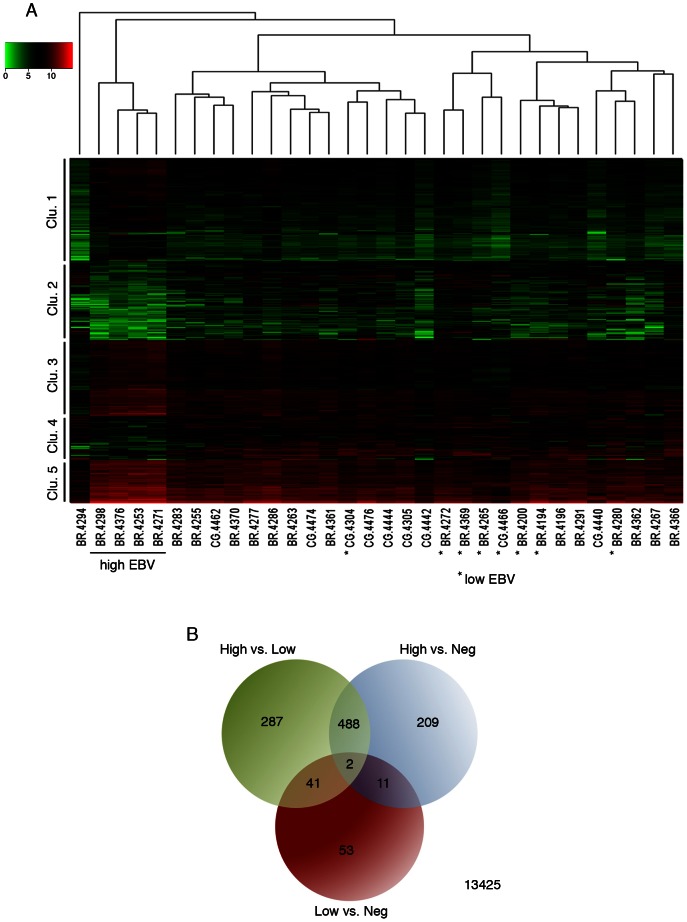
Cluster analysis of EBV-associated gastric carcinoma samples. (A) A representative cohort of 32 gastric carcinoma samples (12 EBV-positive and 20 EBV-negative) were grouped using hierarchical clustering and are displayed with an expression heat map of the 490 genes that were found to be significantly differentially expressed in high EBV. (B) The cohort of 32 gastric carcinoma samples was divided into three categories (high EBV, low EBV, and negative). These categories were subjected to differential gene expression analysis using edgeR. The Venn diagram displays the numbers of all statistically significant differentially expressed genes. Statistical significance was determined by an adjusted *P* value<0.05.

To investigate influences of EBV dependent alterations in tumor signaling pathways, we analyzed global cellular gene expression in all 12 EBV positive specimens plus an additional 20 randomly selected EBV negative samples. EBV gene expression data was not included in this analysis to ensure that clustering occurred based only on differences in cellular gene expression (i.e. that it occurred independently of biases incurred by the presence of EBV gene expression signatures). Strikingly, when the set of samples were analyzed using hierarchical clustering, the four gastric carcinoma samples with higher numbers of EBV reads (BR-4253, BR-4271, BR-4376, and BR-4298) formed its own well-separated group (Figure 7A). One of the EBV negative samples, BR-4294, clustered independently of the others and subsequent analysis revealed that this sample was likely an outlier (Figure S5). Nevertheless, this sample was retained in the subsequent differential expression analysis as a conservative measure.

Human transcript counts from the EBVaGCs with high EBV read levels were compared to the EBVaGCs with low EBV read numbers and with the EBVnGCs. Using this approach, 490 genes were found to have statistically significant differential expression in the “high” EBVaGC (hiEBVaGC) samples relative to both EBVnGC and “low” EBVaGCs (loEBVaGC) samples (Figure 7A–B and Table S2). These genes separated into five distinct clusters with clusters 1, 3, and 5 showing genes that were predominately expressed at higher levels in hiEBVaGCs and clusters 2 and 4 containing genes that were predominantly expressed at lower levels in hiEBVaGCs (Figure 7A). We also performed an additional clustering analysis using only the EBV genes across the 12 EBVaGC. This analysis revealed that the 4 hiEBVaGC samples cluster distinctly from the other EBVaGC samples (Figure S6). This apparently distinct gene expression pattern observed in the 4 hiEBVaGC samples raises the possibility that these samples represent infection of a unique cell type relative to the other samples (possibly tumor cells versus stroma or B-cells).

**Figure 8:**
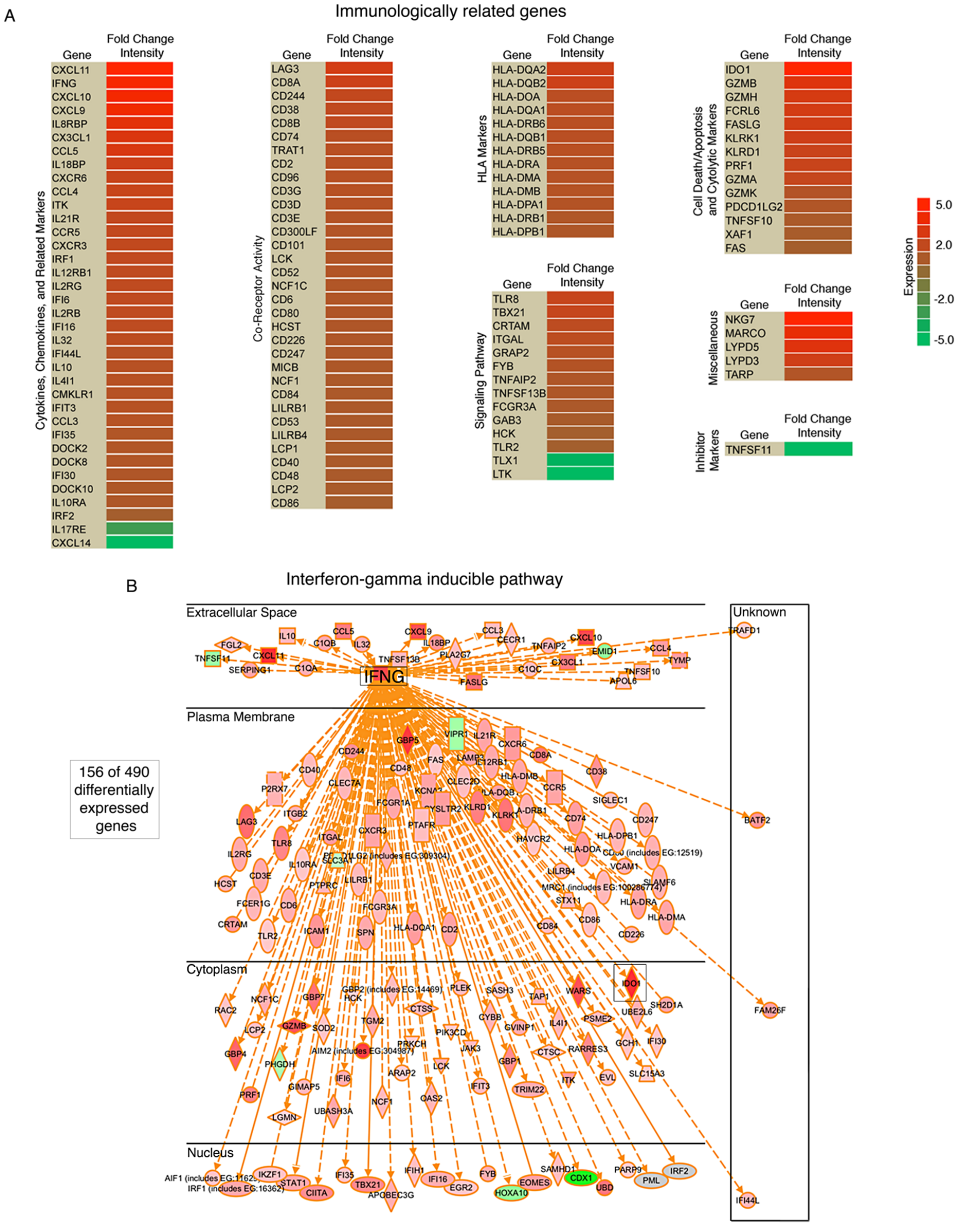
High numbers of infiltrating immune cellular genes are detected in EBVaGC. (A) Significant immunologically related genes differentially expressed in EBVaGC are represented in a heat map. The log^2^ fold change intensities are represented by the color gradient with red corresponding to the highest intensity and green corresponding to the lowest. (B) Interferon-gamma (IFNG) associated genes differentially expressed in EBVaGC are displayed in a diagram.

Ingenuity Pathway Analysis software (IPA: Ingenuity Systems) was used to assist the analysis of pathways and known molecular functions associated with differentially expressed genes. Twenty four percent (116) of the 490 genes with statistically significant differential expression were found to be immunologically related genes (Figure 8A). The vast majority of these genes were expressed at higher levels in hiEBVaGCs with IDO1 and IFNG ranking among the top (38-fold and 16-fold, high v. negative). The differentiation and other cell surface marker profiles are consistent with the presence of cytotoxic T-cells (CTLs) and/or natural killer (NK) cells in hiEBVaGC. Further, CTLs and NK cells are key producers of granzymes and perforin, which are found to be elevated in the hiEBVaGC (Figure 8A).

The interferon gamma (IFNG) pathway was analyzed further using IPA to determine the extent of IFNG pathway involvement in hiEBVaGC. We observed marked involvement of the IFNG pathway with 156 of the 490 differentially expressed genes associated with the IFNG pathway, the majority of which were elevated (Figure 8B).

**Figure 9:**
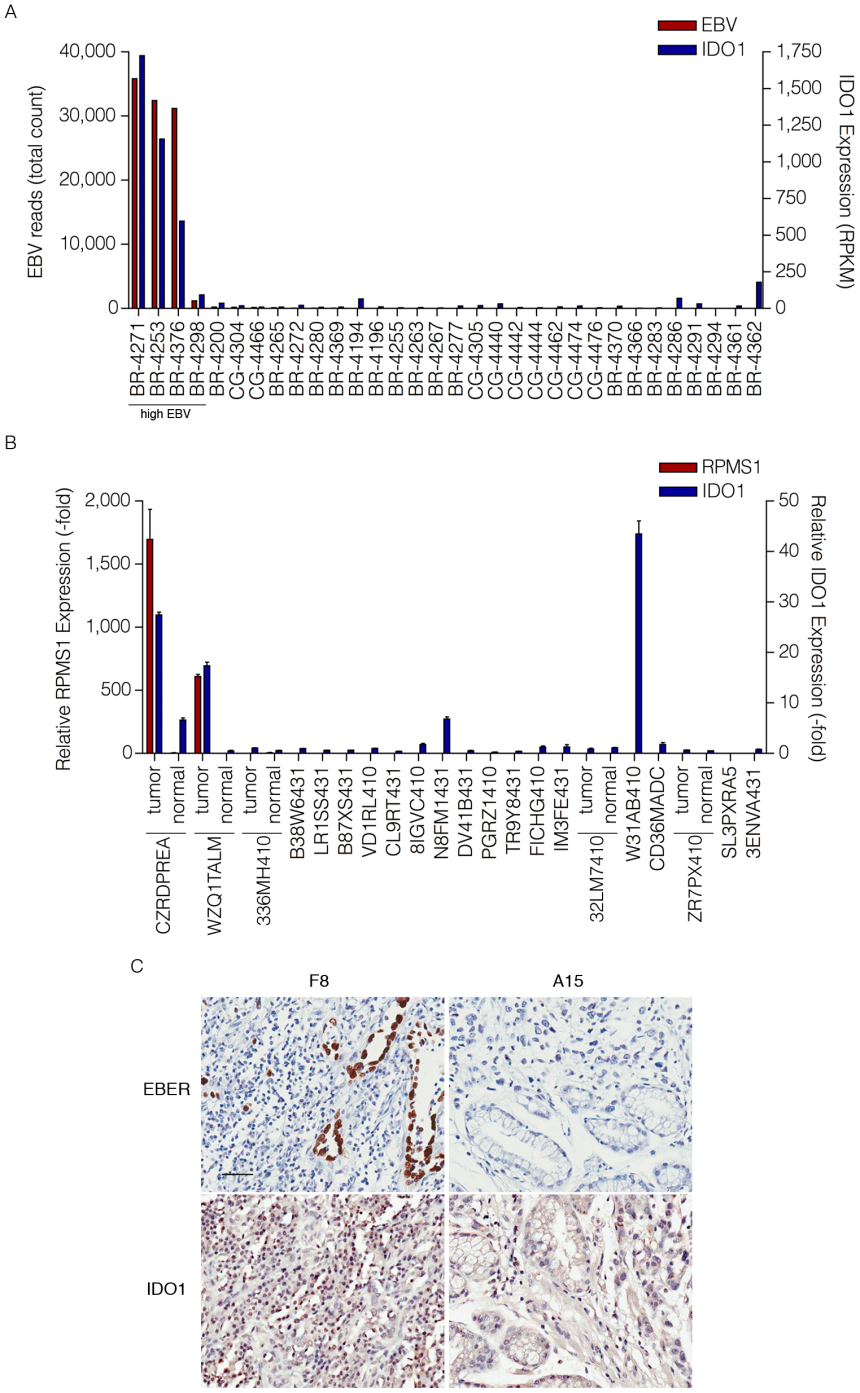
High levels of IDO1 in high EBV positive gastric carcinomas. (A) Gene expression profile of the cohort of 32 gastric carcinoma samples (12 EBV-positive and 20 EBV-negative). Both total EBV reads and IDO1 expression (RPKM-reads per kilobase of exon model per million mapped reads) are represented as red and blue columns, respectively. (B) Gene expression profile of the cohort of 21 Vietnamese gastric carcinomas and 5 normal adjacent samples. Both relative RPMS1 expression (-fold) and relative IDO1 expression (-fold) are represented as red and blue columns and are the fold difference compared to the average of normal adjacent control values. (C) Images of paraffin-embedded human gastric carcinoma probed for EBER using in situ hybridization or IDO1 staining with immunohistochemistry. F8 and A15 each represent a specific gastric carcinoma on the tissue array selected to be closely matched with respect to age, tumor grade and stage. Scale bar represents 50 µm.

The analysis of IDO1 levels for each of the 32 gastric carcinomas showed that the samples with the highest number of EBV reads had the highest levels of IDO1 expression (Figure 9A). To further explore the link between EBV and IDO1, we analyzed a separate cohort of Vietnamese gastric carcinoma samples by real time RT-PCR. RPMS1 was detected in two of these samples (CZRDPREA and WZQ1TALM) (Figure 9B) and these samples ranked among the highest for expression of IDO1 (27 and 17 fold relative to the average of the 5 normal adjacent tissue samples). Further, in these samples, normal adjacent tissue showed lower RPMS1 expression and lower IDO1 expression compared to their tumor counterparts. Notably, one of the EBV negative samples, W31AB410, showed the highest level of IDO1 (43 fold). Nevertheless, this sample was notable in that like the two EBV positive samples, the pathology report for this sample similarly noted high levels of immune cell infiltration which may result from the presence of another infectious agent.

*In Situ Hybridization* for EBER was performed on a gastric carcinoma tissue array (ST2091; US Biomax) in order to assess the presence of EBV. In the strongly EBV positive cases, EBV was detected in the epithelial cells (e.g. F8 in Figure 9C). A high level of immune cell infiltration is observed in EBV positive (e.g. F8, Figure 9C) but not the tumor grade matched EBV negative sample, A15 (Figure 9C) with a high proportion of the immune cells in F8 showing intense IDO1 staining.

Analysis of the 178 down regulated genes showed that 19 tumor suppressor genes and 13 candidate oncogenes were found to be expressed at lower levels in hiEBVaGC (Table S3). Furthermore, we observed several inhibitors of the hedgehog and Wnt pathways to be expressed at lower levels in hiEBVaGCs suggesting additional components to the complex interactions involved in EBVaGC pathogenesis.

## Discussion

Consistent with the Shibata and Weiss study for the incidence of EBVaGC in the United States using ISH against EBERs [2], we detected EBV in 12 of the 71 (17%) gastric carcinoma samples from The Cancer Genome Atlas (TCGA) cohort using RNA CoMPASS. The detection of EBV using EBER ISH is widely used and the similar detection levels between the Shibata and Weiss study [2] and our work suggest that both methods are accurate for determining the presence of EBV in biological specimens. Importantly, however, the use of RNA-seq data allowed us to also infer the magnitude of local environmental signaling influences for different levels of EBV infection/viral gene expression. While the four samples with higher levels of EBV transcripts formed a clearly distinct cellular gene expression cluster, the eight samples with low numbers of EBV reads clustered in a mixed fashion among the EBV negative specimens. We propose that these two classes of EBV infection should be considered functionally distinct with possible implications in therapeutic intervention decisions and/or therapeutic response predictions.

RNA CoMPASS has the potential to simultaneously allow for the investigation of all pathogens present in tumor samples. In addition to EBV, we detected low levels of enterobacteria phage T4T, HCMV, Hepatitis C virus, and *Helicobacter pylori* (data not shown). The detection of enterobacteria phage T4T and Hepatitis C virus transcripts should be met with caution due to the likely possibility of environmental contamination and misclassification of these reads, respectively. While the HCMV reads likely represent true HCMV infection of cells within the tumor sample, the low read levels suggest either low numbers of HCMV infected cells or limited expression of polyadenylated viral RNAs. Finally, we detected *H. pylori* in three of the gastric carcinoma samples but the number of reads was very low in each case. Since bacterial RNAs are typically not polyadenylated or have limited numbers of polyadenylated RNAs [22], [23], [24], [25], this low detection level probably results from the sequencing libraries being prepared from polyA selected RNA rather than an absence of *H. pylori* in these samples.

Of the 12 EBVaGCs, there was sufficient EBV read coverage in four of the samples to carry out more detailed transcriptomic analysis. LMP2, EBNA1, and LMP1 expression was detected in three of the EBVaGCs and these results are generally consistent with the findings of other groups [15], [16], [17], [18]. The magnitude of expression from the BamHI A region relative to the transcription levels of other EBV genes is striking, however. This result is consistent with a previous report using a naturally infected EBV positive gastric carcinoma cell line [26]. Nevertheless, our analysis makes this observation in the natural *in vivo* setting of the tumor, and the use of RNA-seq facilitated the evaluation of transcript structures and the magnitudes of BamHI A region gene expression relative to other viral and cellular genes.

Although others have been unable to detect protein from naturally expressed BamHI A rightward transcripts [27], [28], the high expression level of these transcripts in hiEBVaGC samples suggests a functional role in gastric adenocarcinomas; possibly as long non-coding RNAs (lncRNA). These rightward BamHI A transcripts also encode as many as 44 intronic microRNAs (miRNAs) [29], [30]. The function of the BART miRNAs in the EBV life cycle and in EBV associated malignancies is currently unclear but a recent study by Raab-Traub's group provided evidence that the BART miRNAs contribute to the tumor phenotype in EBVaGC [31]. In Raab-Traub's study, several lines of evidence supported this contention. First, very little EBV latent protein expression was detected and inhibition of the small amount of LMP1 expressed did not affect the cell's phenotype. Second, they observed that the majority of the significant cellular gene expression changes following infection of AGS (a gastric carcinoma cell line) cells with EBV were down regulated, many of which were significantly enriched in both experimentally and bioinformatically predicted BART miRNA targets [31], [32]. Based on this evidence and the fact that the BamHI A rightward transcripts are expressed at high levels in gastric carcinomas, it seems likely that the BART miRNAs play an important role in modulating the cellular phenotype in this tumor type. Nevertheless, many lncRNAs are involved in repressive complexes raising the possibility that the high levels of spliced rightward BamHI A transcripts that we detect *in vivo* may function as lncRNAs which similarly contribute to repression of cellular gene expression in hiEBVaGCs. Our strand specific RNA-seq analysis of SNU-719 cells further support our contention of high level expression of the rightward RPMS1 and A73 related transcripts in gastric carcinomas. This analysis also demonstrated the presence of additional rightward exons/genes within this region that may similarly play a role in lncRNA mediated regulation of viral and/or cellular signaling.

Although EBV primarily exhibits latent gene expression patterns in EBV associated tumors, recent studies using EBV associated lymphoma models suggest that a small portion of tumor cells express lytic transcripts that promote tumor growth [33], [34], [35], [36]. The Kenney lab showed that B cells harboring an EBV BZLF1 knock out mutant grew slower than wild type infected cells in a SCID mouse xenograft model [33]. In a separate study, they showed that a mutant EBV over expressing BZLF1 induces lymphomas with abortive lytic EBV infection in a humanized mouse model [36]. By assessing global EBV gene expression, we provide evidence for an abortive lytic phase *in vivo*; in the context of the natural setting of a human tumor. This supports the lymphoma animal studies from the Kenney group and raises the possibility that an abortive lytic phase may also play a role in EBV associated epithelial tumors.

One EBVaGC sample (BR-4253) was found to express high levels of BNLF2A/B. In the absence of significant expression of other lytic genes, the detection of BNLF2A/B expression in this sample was unexpected. One of the simplest models to explain this observation is a possible viral genetic alteration that juxtaposes this gene with an active viral promoter; in a manner reminiscent of the previously identified hetDNA (BZLF1 gene recombined to an active latency promoter) [37], [38], [39]. Alternatively, this could result from a rare viral integration event positioning the BNLF2A/B gene downstream from an active cellular promoter. Just as advantageous genetic alterations evolve in the cellular genome during cancer progression, a genetic event that resulted in the activation of BNLF2A/B may be an example of an advantageous viral genetic alteration that was selected during tumor evolution. BNLF2A was shown previously to function as an immune evasion protein through HLA class I down regulation (via blocking of TAP activity) [40]. This anti-immune function may have been selected for during tumor evolution and may support viral/tumor survival in this patient.

**Figure 10:**
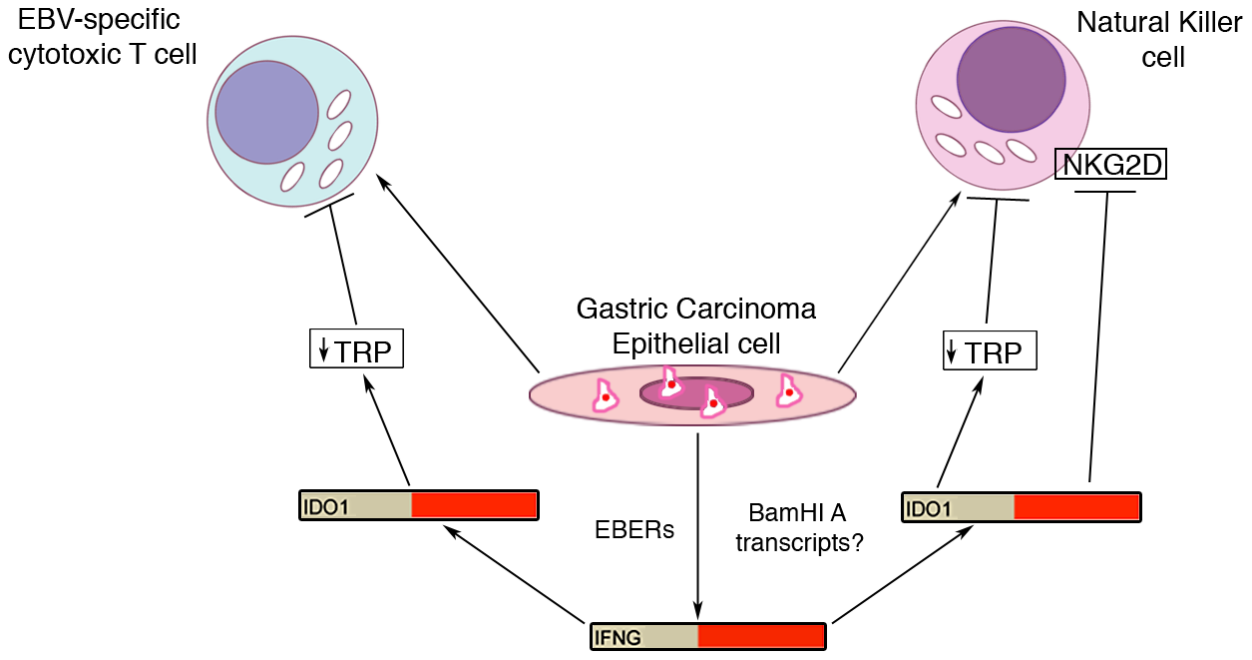
Model for EBV modulation of cytotoxic T-cell and natural killer cell function in tumor microenvironment. EBV infected gastric carcinoma cells recruit cytolytic immune cells such as T-cell and natural killer cells via unclear mechanisms. In addition, these cells induce an increase in interferon-gamma (IFNG) via EBERs and possibly BamHI A transcripts. Increased IFNG results in increased IDO1 resulting in depleted tryptophan. Depleted tryptophan results in T-cell and natural killer cell inhibition.

Cellular RNA expression profiling provided strong evidence for immune cell infiltration in hiEBVaGCs. This can be seen in tissue sections from EBV positive specimens (e.g. see Figure 9C) and is further supported by the pathology reports from the two EBV positive gastric carcinoma samples from the Vietnamese cohort which indicate high levels of immune cells (Table S4). This observation is in line with previous studies using standard hematoxylin and eosin staining of tumor sections [3], [41] where lymphocyte infiltration was found to be predominately CD8+ T cells [42], [43]. Notably, however, despite this apparent robust immune response in hiEBVaGC, EBV and the infected tumor cells are able to persist in these patients. This suggests that these tumors may have compensatory immune evasion strategies that allow virus/tumor survival in this setting [44]. First, the limited expression of viral protein coding genes in EBVaGC likely contributes to the avoidance of viral antigen targeting [45]. Second, although the EBV encoded protein, EBNA1 is required for viral episomal replication/maintenance and therefore must be expressed in proliferating cells, it encodes a glycine-alanine repeat domain that blocks its proteasomal processing for CTL presentation [46], [47]. Third, here we found that expression of the interferon-gamma (IFNG) inducible CTL and NK inhibitor, indoleamine 2,3-dioxygenase (IDO1) is high in hiEBVaGC. IDO1 is a rate-limiting enzyme involved in the catabolism of tryptophan (Trp) [48]. CTLs and NK cells are uniquely sensitive to Trp depletion leading to the induction of stress responses and the inhibition of proliferation and activation [49], [50]. IDO1 functions to cause local tryptophan depletion under physiological and pathogenic immune tolerance settings such as during placentation and cancer [51], [52] where it is considered to be critical for establishing local immune tolerance. Among other candidate effectors, increased IFNG has been shown to induce IDO1 expression [53], [54]. Therefore, despite the apparent increase in CTL and NK cells in hiEBVaGCs, the activated IFNG signaling may counteract this response through IDO1 mediated Trp depletion (Figure 10); allowing tumor survival.

The findings of high IDO1 levels in several cancers and studies showing that IDO1 is critical for tumor survival has led to intense interest in the potential of anti-IDO1 based immunomodulatory therapeutics [55], [56], [57], [58]. IDO1 inhibitors, such as the small molecule inhibitor, 1MT, have shown anti-tumor potential in combination with conventional chemotherapeutic drugs [57], [58]. This raises the important possibility that the therapeutic response for at least the subset of hiEBVaGCs may similarly be enhanced by the addition of IDO1 targeting therapeutics.

In our study, 156 of the genes found to be differentially expressed in EBVaGCs are linked to the IFNG pathway. The EBV encoded small RNAs, EBERs, have been shown to induce the expression of IFNG [59], and they likely play a significant role in the active IFNG response observed here. Despite this, the extensive level of secondary structure guiding the processing of the BamHI A rightward introns during the miRNA processing steps may similarly contribute to the IFNG response in EBVaGCs observed here (Figure 10).

EBVaGCs exhibit extensive nonrandom DNA methylation at the promoter regions of various cancer-related genes [60], [61] and has been classified as having the CpG island methylator phenotype (CIMP) [62]. Several studies have investigated possible mechanisms of promoter hypermethylation of host genes in association with EBV infection. LMP1 mediated activation of DNA methyltransferase 1 (DNMT1), through either activation of c-Jun NH_2_-terminal kinase (JNK)-activator protein-1 (AP-1) signaling [63] or through RB-E2F pathway activation [64], have been proposed as mechanisms in some systems. However, EBVaGCs do not typically express significant levels of LMP1. A study by Hino et al. demonstrated DNMT1 activation via LMP2A [65] raising the possibility that a LMP2A/DNMT1 mechanism could be involved. Nevertheless, a study by Chong et al. showed that DNMT expression was suppressed in EBVaGC and that the methylation of specific genes occurs through a mechanism independent of DNMT1 activation [66]. Based on this observation and on our findings of relatively low levels of LMP1 and LMP2A expression in EBVaGCs, we propose that methylation/imprinting may be downstream of more direct EBV inhibitory mechanisms. The robust expression levels of the BamHI A transcripts in EBVaGCs put them high on the radar as candidates for this type of regulation, possibly through lncRNA mediated chromatin imprinting based mechanisms.

Multiple tumor suppressors were expressed at lower levels in EBVaGCs including five (TFF2, RBP4, HOXA9, LRRN1, and RAP1GAP) that are known to be hypermethylated in cancers [67], [68], [69], [70], [71]. Another gene expressed at lower levels in EBVaGCs was HNF4A, a cell-specific transcription factor known to regulate a large number of genes in liver, intestine, pancreas, and stomach [72]. Decreased expression of HNF4A has been shown in renal cell carcinoma [73] and has recently been shown to regulate key genes involved in cellular proliferation [72]. A recent study by Lucas and colleagues suggest that HNF4A acts as a tumor suppressor [72].

In addition to tumor suppressors, we also observed several candidate oncogenes to be expressed at lower levels in EBVaGCs including 4 (CDH17, CDX1, ETV4, and PPP1R1B) known to be over expressed in gastric carcinoma and gastrointestinal cancers [74], [75], [76], [77]. Although EBV clearly contributes to cancers, its oncogenic properties are a byproduct of its life cycle rather than an evolved tumor promoting function. In line with this concept, the lower levels of these oncogenes in EBVaGCs may be a byproduct of EBV's life cycle. Conversely, it is possible that the non-EBV mediated gastric carcinoma oncogenic pathway occurs through the up-regulation of these genes whereas the EBV assisted oncogenic path does not. Regardless of which of these principles may explain this observation, the lower levels of oncogenes in EBVaGC may partly explain the more favorable prognosis that is often observed in EBVaGC. Similarly, the lower levels of USP2, a negative regulator of p53, may help explain the normal to elevated levels of p53 found in EBVaGC [78], [79] and possibly the better responses to chemotherapeutics.

An increase in sonic hedgehog (SHH) expression and its activation in gastric carcinoma, especially *H. pylori* associated gastric carcinomas has been well established [80]. In our study, several inhibitors of both the SHH and Wnt pathways were found to be lower in hiEBVaGC including HHIP (SHH) and SHISA3, NKD2 and LRP4 (Wnt). The decrease in SSH inhibitor, HHIP, [81] suggests that Hedgehog activity may be higher in hiEBVaGC. Down regulation of HHIP in pancreatic cancer has been shown to be mediated through epigenetic CpG hypermethylation within the promoter region [82]. This raises the possibility of a specific methylation process by EBV, since we observe a significantly lower level of HHIP reads in the hiEBVaGC compared to loEBVaGC and EBVnGC. Hypermethylation of the promoter region of NKD2 has been established in malignant astrocytic gliomas [83], and a CpG island within the SHISA3 and LRP4 promoter regions have been identified [84]. This suggests that epigenetic silencing of these Wnt pathway inhibitors may also occur through an EBV mediated mechanism.

## Materials and Methods

### Clinical tumor sample and sequence data acquisition

All human specimens were de-identified prior to acquisition. Total RNA from 21 Vietnamese gastric carcinoma samples and 5 normal adjacent samples were obtained from Biospecimen Repository at Bioserve (Beltsville, MD). Demographic and clinical data is available in table S4. RNA-seq data from 71 gastric carcinoma samples generated through the National Institutes of Health, The Cancer Genome Atlas (TCGA) project were obtained from the NCBI Sequence Read Archive (SRA035410, now available through the Cancer Genomics Hub managed by the University of California, Santa Cruz (UCSC)). Demographic and clinical data for each sample is available through the TCGA data portal (http://cancergenome.nih.gov/). Briefly, samples were obtained from non-Hispanic White Russians with no previous treatment. The mean age was 69 years with a range of 43 to 90 years. Total RNA was isolated from each sample using mirVana RNA kit according to TCGA. High quality RNA was polyA selected and sequenced using an Illumina Genome Analyzer II machine running paired end 51 base sequencing reactions with two lanes of sequence per sample.

### Cell culture

SNU-719 gastric adenocarcinoma cells were obtained from the Korean Cell Line Bank. They were grown in RPMI 1640 (Thermo Scientific; Waltham, MA) plus 10% fetal bovine serum (Invitrogen-Gibco; Grand Island, NY) with 1% penicillin-streptomycin (Invitrogen-Gibco; Grand Island, NY). Cells were grown at 37°C in a humidified, 5% CO_2_ incubator.

### Sample preparation and next generation DNA sequencing

Total RNA was extracted from SNU-719 cells using the miRNeasy Mini Kit (Qiagen, Hilden, Germany) according to manufacturer's instructions. Two separate cDNA libraries were prepared from polyA selected and from Ribo-Zero selected RNAs using the Illumina Truseq Stranded Total RNA Sample Prep Kit (RS-122-2101). 101-base paired-end sequencing was performed using an Illumina HiSeq 2000 instrument. The SNU-719 RNA-seq data used in this publication have been deposited in NCBI's Gene Expression Omnibus [85] and are accessible through GEO Series accession number GSE45453 (http://www.ncbi.nlm.nih.gov/geo/query/acc.cgi?acc=GSE45453).

### RNA CoMPASS

RNA CoMPASS (Xu et al., unpublished) a graphical user interface (GUI) based parallel computation pipeline, RNA comprehensive multi-processor analysis system for sequencing (RNA CoMPASS) for the analysis of both exogenous and human sequences from RNA-seq data (Figure S1). Briefly, for the analysis of both exogenous and human sequences, raw sequence data is first processed through an in house de-duplication algorithm. Following de-duplication, reads are aligned to a reference genome containing human (hg19; UCSC) and abundant sequences (which include sequence adapters, mitochondrial, ribosomal, enterobacteria phage phiX174, poly-A, and poly-C sequences). Novoalign V2.07.18 (www.novocraft.com) [-o SAM, default options] is used to map reads to the reference genome and to eliminate low-quality reads (QC<20). TopHat V1.4.0 [default options] [21] is used to identify and isolate all sequences that map to human splice junctions. The results from these programs are compiled and separated into mapped reads (used for human transcriptome analysis) and unmapped reads (used for exogenous sequence analysis). Human mapped reads are analyzed using SAMMate [13] to quantify gene expression and to generate genome coverage information. Unmapped reads are subjected to consecutive BLAST V2.2.24 searches against the Human RefSeq RNA database (an additional “pre-clearing” step) and then to the NCBI NT database to identify reads corresponding to known exogenous organisms [86]. Results from the NT BLAST searches are filtered to eliminate matches with an E-value of less than 10e^−6^. The results are fed into MEGAN 4 [87] for convenient visualization and taxonomic classification of BLAST search results.

RNA CoMPASS is designed to take advantage of parallel processing at several key steps to speed processing times. In our case, we used a four node, 12 core, Intel Xeon Mac Pro (64GB of memory per node) cluster.

### Human and EBV transcriptome quantification

Samples containing evidence of EBV were identified using RNA CoMPASS. Since each sample contained sequence data from two runs, data from both runs were combined in order to generate a greater sequencing depth for transcript quantification. In addition, 20 EBV negative samples were randomly chosen for analysis. Samples were aligned to a reference genome containing human (hg19) and a modified EBV B95-8 genome that contains Raji genome sequences inserted into a deleted region of the B95-8 genome (Genbank accession number AJ507799) using Novoalign V2.07.18 (www.novocraft.com) [-o SAM, default options]. Transcript data from Novoalign was analyzed using SAMMate for transcript quantification of human and EBV genes and to generate coverage (wiggle) files for visualization of read distributions. Splice junction data was generated using the junction aligner, TopHat V1.4.0 [default options]. Coverage data was visualized using the Integrative Genomics Viewer (IGV) [88].

### Quantitative RT-PCR

Total RNA was reverse-transcribed using the SuperScript III First-Strand Synthesis System for RT-PCR (Invitrogen, Carlsbad, CA). Random hexamers were used along with 250 ng RNA in a 20 µl reaction volume according to manufacturer's instructions. For the incubation steps (25°C for 10 min followed by 50°C for 50 min) a Mastercycler ep (Eppendorf, Hamburg, Germany) was used. For real-time PCR, 1 µl of the resulting cDNA was used in a 10 µl reaction mixture that included 5 µl of 10× SsoFast EvaGreen supermix (Bio-Rad, Hercules, CA), 1 µl of 10 µM forward and reverse primer mix (Integrated DNA Technologies, Coralville, IA), and 3 µl of PCR grade water. The IDO1 primers amplified a 112 base pair product. Forward primer 5′-CAAATCCACGATCATGTGAACC-3′ and reverse primer 5′-AGAACCCTTCATACACCAGAC-3′ were used previously by Prachason et al [89]. The RPMS1 primers amplified a 181 base pair product consisting of exon 6 and exon 7. Forward primer 5′-CCAGGTCAAAGACGTTGGAG-3′ and reverse primer 5′-CACCACGGTGCAGCCTAC-3′ were used. The GAPDH primers amplified a 297 base pair product. Forward primer 5′- CAATGACCCCTTCATTGACC-3′ and reverse primer 5′- GACAAGCTTCCCGTTCTCAG-3′ were used. Each sample was performed in triplicates. No-template controls and no-reverse transcription controls were also included in each PCR run. Thermal cycling was performed on a CFX96 Real Time System (Bio-Rad, Hercules, CA) and data analysis was performed using the CFX Manager 3.0 software. Cycling conditions included an initial incubation at 95°C for 30 seconds followed by 40 cycles consisting of 95°C for 5 seconds, and 60°C for 5 seconds. Melting curve analysis was performed at the end of every qRT-PCR run.

### In-situ hybridization

Chromogenic In Situ Hybridization (CISH) was performed by the Tulane Molecular Pathology Lab using the HistoSonda EBER probe kit (American Master Tech, Lodi, CA) according to manufacture's instructions. The tissue array was deparaffinized and rehydrated in a graded solution of Xylene and alcohol. Tissue array was deproteinized using Proteinase K and incubated with Digoxigenin EBER probe. Tissue array was subsequently washed with water and PBS. The tissue array was incubated with Anti-digoxin and anti-mouse horse radish peroxidase to form a duplex with the Digoxigenin EBER probe. For colorimetric staining, slides were then incubated in 3,3′-Diaminobenzidine (DAB; Vector Laboratories), washed with dH_2_O, counterstained with hematoxylin, and rinsed with PBS (pH 7.4). Slides were dehydrated in a graded solution of alcohol and Xylene and sealed with Permount Mounting Medium (Sigma). To visualize the tissue array, slides were scanned into ScanScope CS2 (Aperio, Vista, CA) and images were acquired with ImageScope (Aperio).

### Immunohistochemistry

Formalin-fixed, paraffin-embedded (FFPE) gastric tumor tissue array (ST2901) was purchased from U.S. BioMax (Rockville, MD). Demographic and clinical data can be found on the U.S. BioMax website (http://www.biomax.us/tissue-arrays/Stomach/ST2091). The tissue array was deparaffinized, and rehydrated in a graded solution of Sub-X clearing medium (Leica Biosystems, Buffalo Grove, IL). Antigen retrieval was performed with Tris-EDTA Buffer, consisting of 10 mM Tris Base, 1 mM EDTA Solution, and 0.5% Tween 20 (pH 9.0), for 30 minutes. The tissue array was then quenched with 3% H_2_O_2_ (Sigma), rinsed with TNT washing buffer made of 0.1 M Tris-HCl, 0.15 M NaCl, and 0.5% Tween-20 (pH 7.5), blocked with blocking reagent purchased from Perkin Elmer (Waltham, MA) and stained with goat-anti-human IDO (Abcam, Cambridge, MA) overnight at 4°C. Tumor sections were subsequently washed in TNT, incubated with donkey-anti-goat HRP conjugated secondary antibody (Santa Cruz, Dallas, TX) for 1 hour at room temperature, and washed with TNT. For colorimetric staining, slides were then incubated in 3,3′-Diaminobenzidine (DAB; Vector Laboratories), washed with dH_2_O, counterstained with hematoxylin, and rinsed with PBS (pH 7.4). Slides were dehydrated in a graded solution of Sub-X clearing medium and sealed with Permount Mounting Medium (Sigma). To visualize the tissue array, slides were scanned into ScanScope CS2 (Aperio, Vista, CA) and images were acquired with ImageScope (Aperio).

### Statistics and cluster analysis

Transcript counts were imported into the R software environment and analyzed using the edgeR package [90]. Genes with low transcript counts (less than 1 CPM (count per million)) in the majority of samples were filtered. The Manhattan (L-1) distance matrix for the samples was computed using the remaining transcript counts, and this was taken as input for hierarchical clustering using the Ward algorithm. The well separated cluster of four EBV positive samples were found to be those with the highest numbers of EBV reads and were classified as “high EBV”. The remaining samples were then classified as “EBV-negative” or “low EBV”. The glmFit function was then used to fit the mean log(CPM) for each group and likelihood ratio tests were used to identify those genes that were differentially expressed in any of the three possible comparisons, with adjusted *p*<0.05 following the Benjamini-Hochberg correction for multiple testing. The fitted log(CPM) values for the subset of genes that were differentially expressed in the high EBV samples relative to both the low EBV and EBV-negative samples were then clustered using the Euclidean distance and complete linkage algorithm to detect groups of co-expressed genes. The EBV transcript counts from all positive samples were imported in MeV [91] for hierarchical clustering using the Manhattan distance matrix and average linkage clustering algorithm.

## Supporting Information

Figure S1 Schematic of RNA CoMPASS (RNA comprehensive multi-processor analysis system for sequencing). RNA CoMPASS is a web browser GUI (graphical user interface) based computational pipeline designed for the analysis of both human and exogenous sequences in RNA-seq data. Briefly, for the analysis of both exogenous and human RNA-seq data, raw sequence data is first processed through an in house de-duplication algorithm. Following de-duplication, reads are aligned to a reference genome containing human (hg19; UCSC) and abundant sequences, which include sequence adapters, mitochondrial, ribosomal, enterobacteria phage phiX174, poly-A, and poly-C sequences. Novoalign V2.07.18 (www.novocraft.com) [-o SAM, default options] is used to map reads to the reference genome and to eliminate low-quality reads (QC<20). In addition, TopHat V1.4.0 [default options] [21] is used to identify and isolate all sequences that map to human splice junctions. The results from these programs are compiled and separated into mapped reads (used for human transcriptome analysis) and unmapped reads (used for exogenous sequence analysis). Mapped reads are analyzed using SAMMate [13] to quantify gene expression and to generate genome coverage information. Unmapped reads are subjected to consecutive BLAST searches against the Human RefSeq RNA database (an additional “pre-clearing” step) and then to the NCBI NT database to identify reads corresponding to known exogenous organisms. Results from the NT BLAST searches are filtered to eliminate matches with an E-value less than 10e-6. The results are input into MEGAN 4 to generate MEGAN files [87] for convenient visualization and taxonomic classification of BLAST search results.
(TIF)

Figure S2 EBV reads per 1,000,000 human mapped reads are displayed.
(TIF)

Figure S3 Non-log (i.e. linear) circos read coverage plot illustrating EBV read coverage for samples with highest EBV read counts. The reference genome used here is the modified B95-8 genome containing Raji genome sequences (Genbank accession number AJ507799). Circular read coverage graphs display the number of reads mapping to each nucleotide position of the genome. Coverage graphs are represented using a linear scale. Note that alignments were performed using a genome that was split between the BBLF2/3 and the BGLF3.5 lytic genes rather than at the terminal repeats to accommodate coverage of splice junctions for the latency membrane protein, LMP2. The terminal repeat region is indicated in the lower right quadrant of the graph and represent the ends of the linear EBV genome.
(TIF)

Figure S4 Expandable log circos read coverage plot illustrating EBV read coverage for samples with the highest EBV read counts. The reference genome used here is the modified B95-8 genome containing Raji genome sequences (Genbank accession number AJ507799). Circular read coverage graphs display the number of reads mapping to each nucleotide position of the genome. Coverage graphs are represented using a log scale. Note that alignments were performed using a genome that was split between the BBLF2/3 and the BGLF3.5 lytic genes rather than at the terminal repeats to accommodate coverage of splice junctions for the latency membrane protein, LMP2. The terminal repeat region is indicated in the lower right quadrant of the graph and represents the ends of the linear EBV genome.
(TIF)

Figure S5 Multidimensional scaling reveals sample BR-4294 is remarkably different than the other samples analyzed. The four high EBV samples are boxed in red.
(TIF)

Figure S6 Cluster analysis of EBV genes from EBV-associated gastric carcinoma samples. The EBV genes from the 12 EBVaGC samples were subjected to hierarchical clustering and displayed with an expression heat map of all EBV genes.
(TIF)

Table S1 Misclassified Hepatitis C virus reads. Reads were extracted from the Hepatitis C virus taxon from sample BR-4298 and subjected to further analysis by manual BLASTN using the NT database (NCBI). Highest ranking human and hepatitis C virus hits are shown.
(XLS)

Table S2 Differential expression data of gastric carcinoma samples using EdgeR.
(XLS)

Table S3 Representative genes with decreased expression in EBVaGC relative to EBVnGC.
(DOC)

Table S4 Clinical and demographic information for Vietnamese gastric carcinoma samples.
(XLS)
