# Supplementary figures and images for: Differences in Gastric Carcinoma Microenvironment Stratify According to EBV Infection Intensity: Implications for Possible Immune Adjuvant Therapy
Source: PLoS Pathog. 2013 May 9;9(5):e1003341. doi: 10.1371/journal.ppat.1003341 (PMC3649992; doi:10.1371/journal.ppat.1003341)

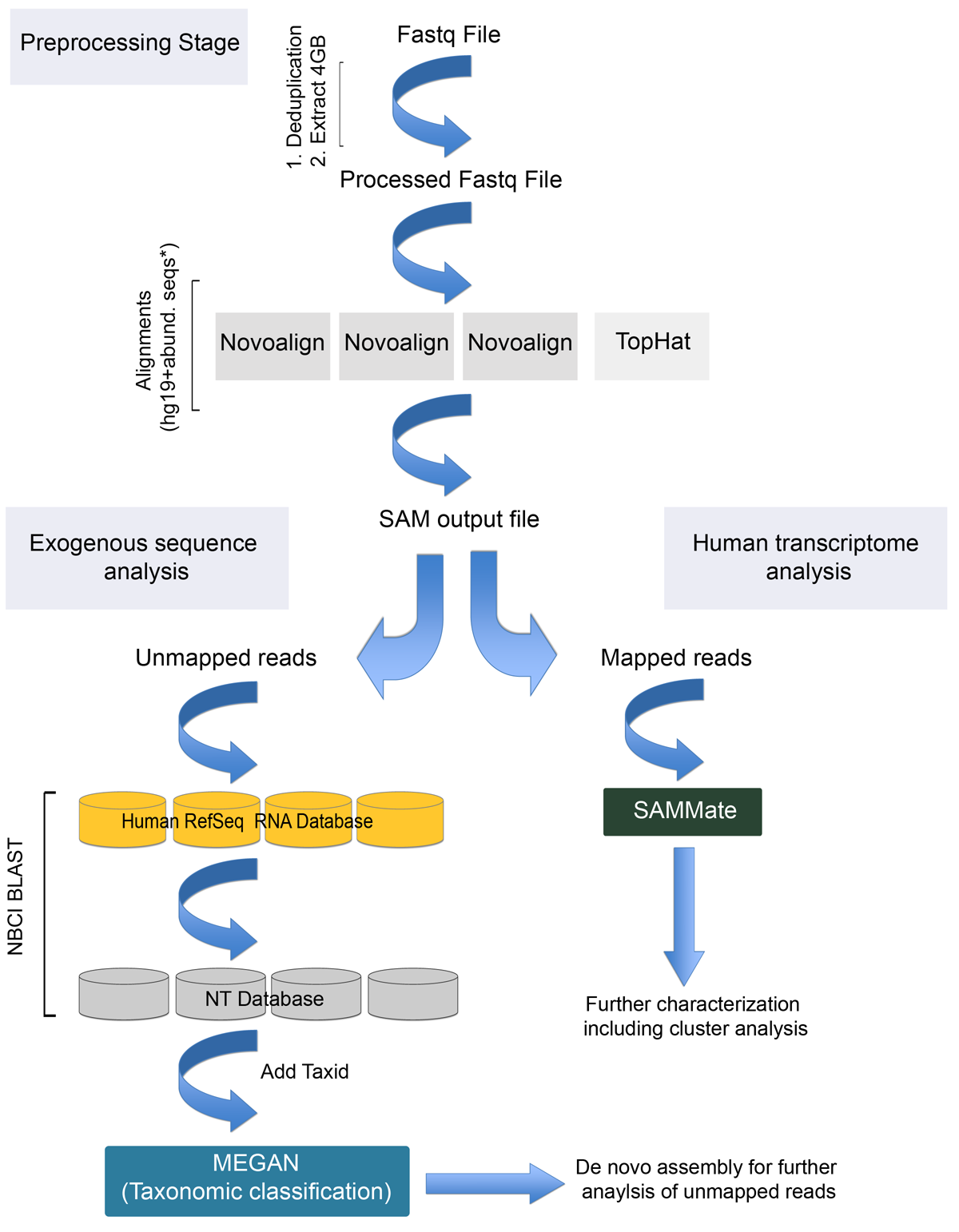

Supplement: Figure S1 — Schematic of RNA CoMPASS (RNA comprehensive multi-processor analysis system for sequencing). RNA CoMPASS is a web browser GUI (graphical user interface) based computational pipeline designed for the analysis of both human and exogenous sequences in RNA-seq data. Briefly, for the analysis of both exogenous and human RNA-seq data, raw sequence data is first processed through an in house de-duplication algorithm. Following de-duplication, reads are aligned to a reference genome containing human (hg19; UCSC) and abundant sequences, which include sequence adapters, mitochondrial, ribosomal, enterobacteria phage phiX174, poly-A, and poly-C sequences. Novoalign V2.07.18 (www.novocraft.com) [-o SAM, default options] is used to map reads to the reference genome and to eliminate low-quality reads (QC<20). In addition, TopHat V1.4.0 [default options] [21] is used to identify and isolate all sequences that map to human splice junctions. The results from these programs are compiled and separated into mapped reads (used for human transcriptome analysis) and unmapped reads (used for exogenous sequence analysis). Mapped reads are analyzed using SAMMate [13] to quantify gene expression and to generate genome coverage information. Unmapped reads are subjected to consecutive BLAST searches against the Human RefSeq RNA database (an additional “pre-clearing” step) and then to the NCBI NT database to identify reads corresponding to known exogenous organisms. Results from the NT BLAST searches are filtered to eliminate matches with an E-value less than 10e-6. The results are input into MEGAN 4 to generate MEGAN files [87] for convenient visualization and taxonomic classification of BLAST search results. (TIF) [file ppat.1003341.s001.tif]

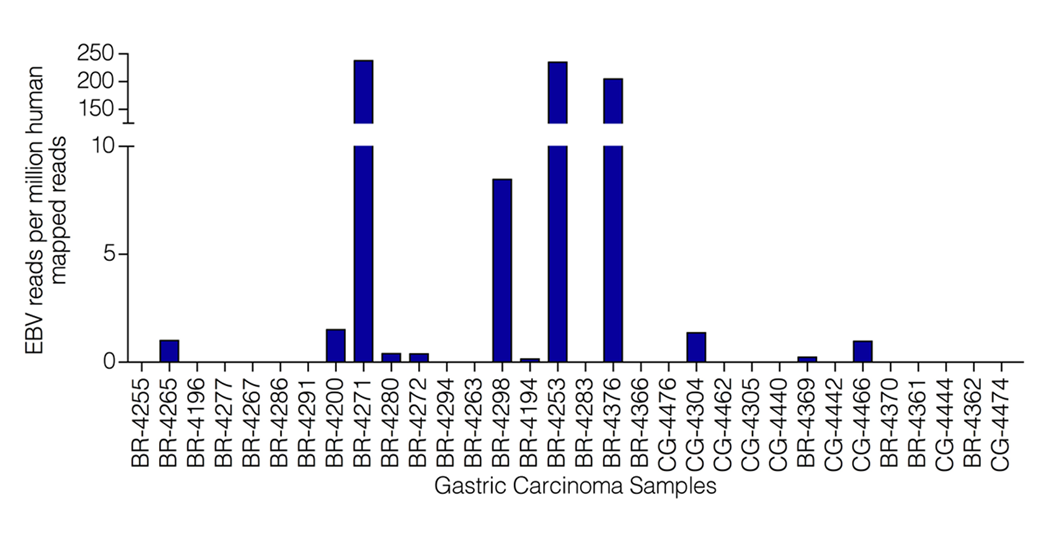

Supplement: Figure S2 — EBV reads per 1,000,000 human mapped reads are displayed. (TIF) [file ppat.1003341.s002.tif]

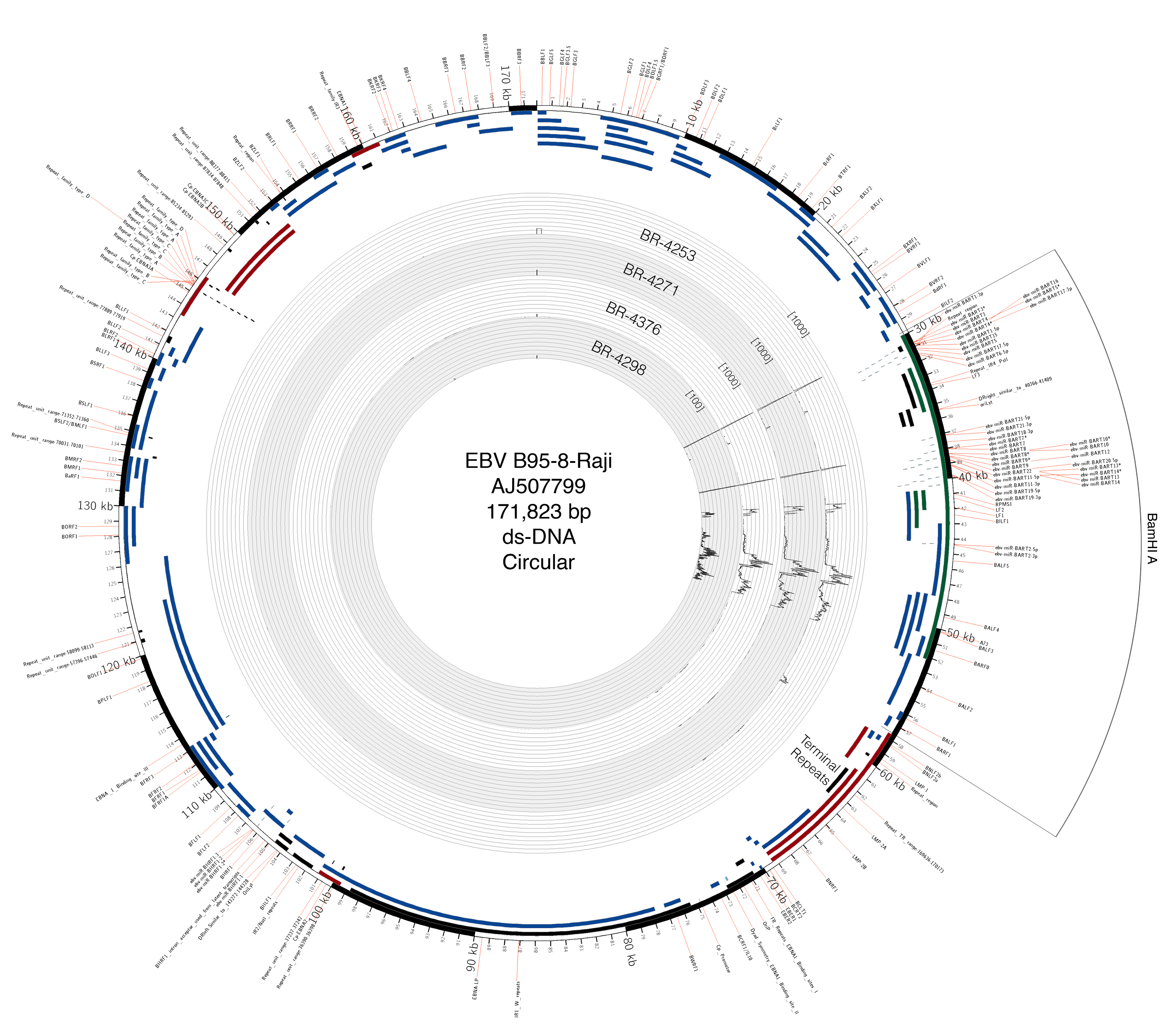

Supplement: Figure S3 — Non-log (i.e. linear) circos read coverage plot illustrating EBV read coverage for samples with highest EBV read counts. The reference genome used here is the modified B95-8 genome containing Raji genome sequences (Genbank accession number AJ507799). Circular read coverage graphs display the number of reads mapping to each nucleotide position of the genome. Coverage graphs are represented using a linear scale. Note that alignments were performed using a genome that was split between the BBLF2/3 and the BGLF3.5 lytic genes rather than at the terminal repeats to accommodate coverage of splice junctions for the latency membrane protein, LMP2. The terminal repeat region is indicated in the lower right quadrant of the graph and represent the ends of the linear EBV genome. (TIF) [file ppat.1003341.s003.tif]

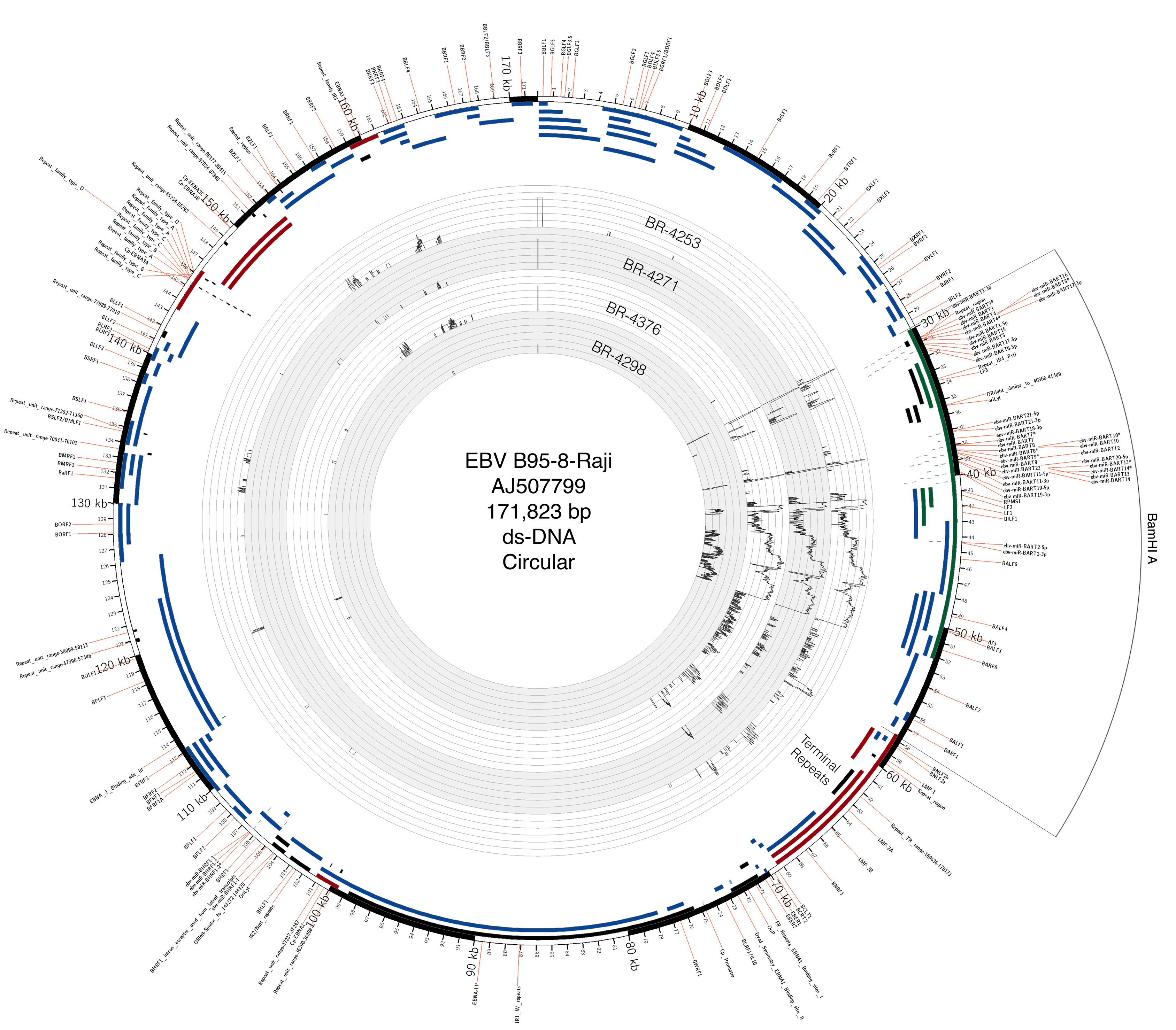

Supplement: Figure S4 — Expandable log circos read coverage plot illustrating EBV read coverage for samples with the highest EBV read counts. The reference genome used here is the modified B95-8 genome containing Raji genome sequences (Genbank accession number AJ507799). Circular read coverage graphs display the number of reads mapping to each nucleotide position of the genome. Coverage graphs are represented using a log scale. Note that alignments were performed using a genome that was split between the BBLF2/3 and the BGLF3.5 lytic genes rather than at the terminal repeats to accommodate coverage of splice junctions for the latency membrane protein, LMP2. The terminal repeat region is indicated in the lower right quadrant of the graph and represents the ends of the linear EBV genome. (TIF) [file ppat.1003341.s004.tif]

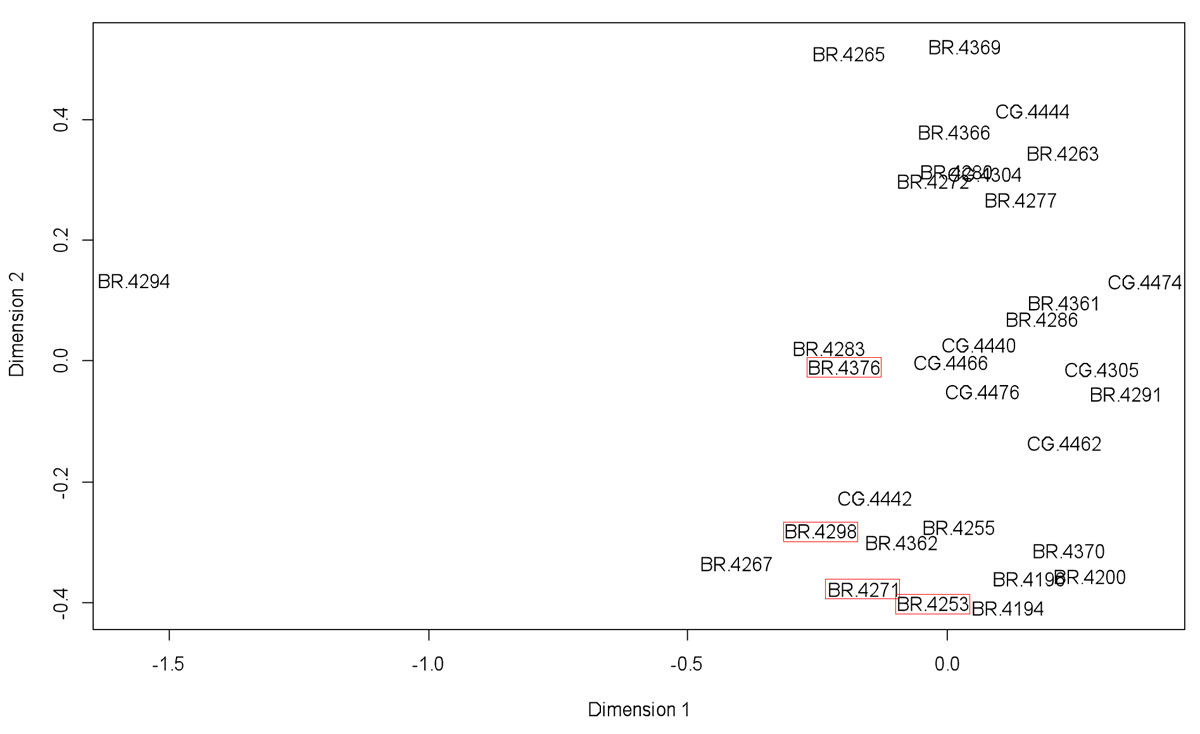

Supplement: Figure S5 — Multidimensional scaling reveals sample BR-4294 is remarkably different than the other samples analyzed. The four high EBV samples are boxed in red. (TIF) [file ppat.1003341.s005.tif]

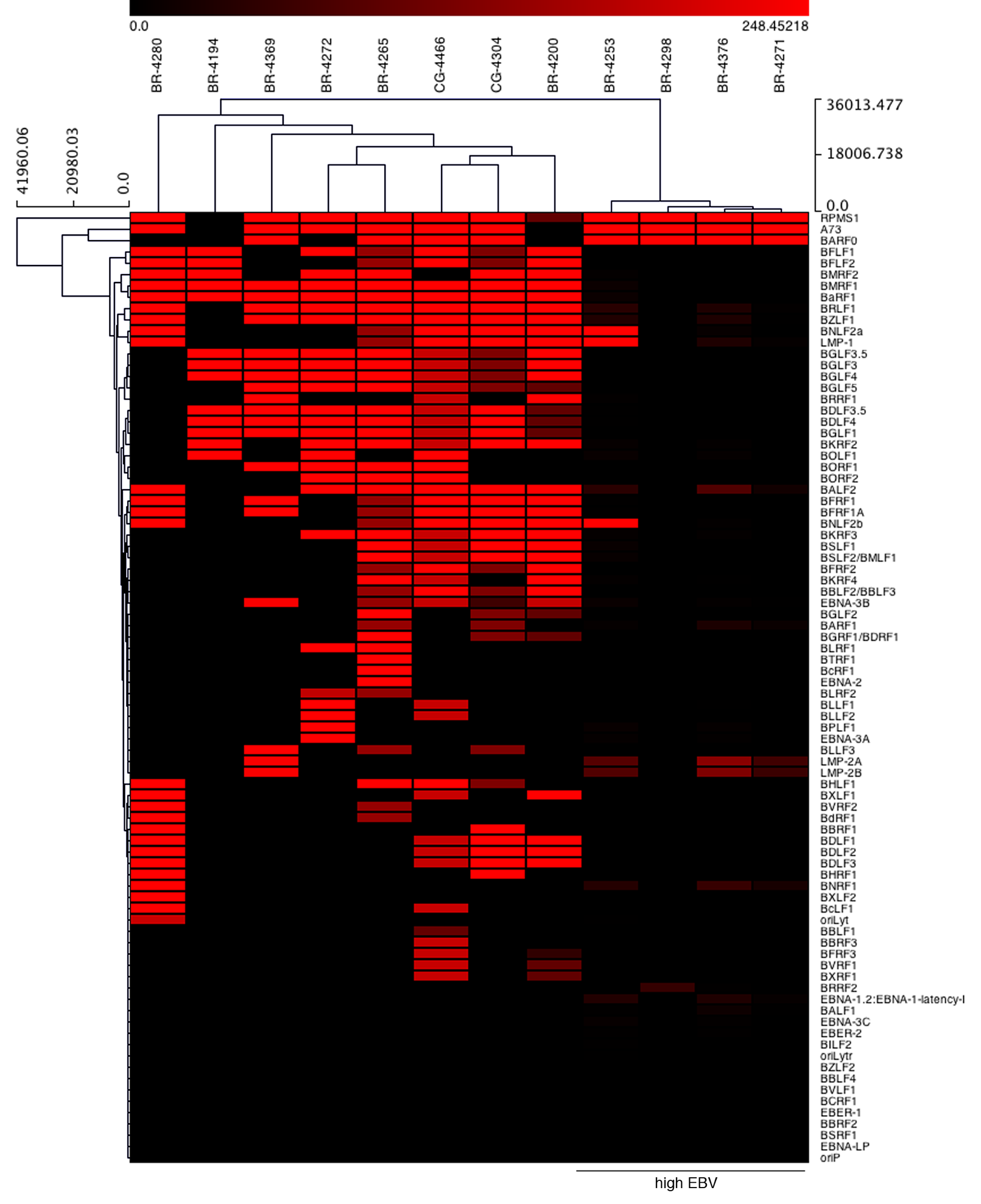

Supplement: Figure S6 — Cluster analysis of EBV genes from EBV-associated gastric carcinoma samples. The EBV genes from the 12 EBVaGC samples were subjected to hierarchical clustering and displayed with an expression heat map of all EBV genes. (TIF) [file ppat.1003341.s006.tif]
