# Supplementary material for: Differences in Gastric Carcinoma Microenvironment Stratify According to EBV Infection Intensity: Implications for Possible Immune Adjuvant Therapy
Source: PLoS Pathog. 2013 May 9;9(5):e1003341. doi: 10.1371/journal.ppat.1003341 (PMC3649992; doi:10.1371/journal.ppat.1003341)
Supplement: Table S3 — Representative genes with decreased expression in EBVaGC relative to EBVnGC. (DOC) [file ppat.1003341.s009.doc]

**Table S3** Representative genes with decreased expression in EBVaGC relative to EBVnGC

| Gene | Log Fold | *P*-value | Function | Refs. |
| --- | --- | --- | --- | --- |
| Tumor Suppressors | | | | |
| GKN2 | -8.1 | 4.70E-02 | Down regulated in gastric carcinoma |  |
| TFF2 | -6.9 | 2.91E-02 | Hyper-methylated in gastric carcinoma |  |
| EFNA2 | -4.8 | 1.31E-04 | Tumor suppressor in gastrointestinal cancers |  |
| CLDN3 | -3.9 | 1.40E-02 | Down regulated in gastric carcinoma leads to proliferative potential |  |
| HOXA10 | -3.4 | 1.31E-02 | Up regulation in gastric carcinoma results in favorable prognosis |  |
| PTCH1 | -2.6 | 1.94E-02 | Tumor suppressor in medulloblastoma |  |
| CNTNAP2 | -4.1 | 6.90E-04 | Acts as tumor suppressor in glioma |  |
| SCARA3 | -2.9 | 3.14E-02 | Tumor suppressor in prostate cancer |  |
| WNK2 | -3.5 | 4.51E-03 | Tumor suppressor |  |
| VIPR1 | -2.9 | 5.19E-03 | Candidate tumor suppressor |  |
| REEP6 | -2.5 | 2.38E-02 | Tumor suppressor |  |
| B3GALT5 | -6.5 | 9.86E-04 | Down regulated in colon cancer |  |
| RBP4 | -5.1 | 3.83E-03 | Hyper-methylated in esophageal carcinoma |  |
| SORBS2 | -2.7 | 2.44E-02 | Down regulated in pancreatic cancer |  |
| HOXA9 | -4.9 | 1.51E-02 | Hyper-methylated in lung cancer |  |
| LRRN1 | -4.5 | 3.09E-03 | Hyper-methylated in non-small cell lung cancer |  |
| FOXA2 | -2.8 | 1.45E-03 | Tumor suppressor in lung cancer |  |
| HNF4A | -1.9 | 1.67E-03 | Candidate tumor suppressor |  |
| RAP1GAP | -2.7 | 1.88E-02 | Hyper-methylated in thyroid cancer |  |
|  |  |  |  |  |
| Oncogenes | | | | |
| CDH17 | -3.7 | 2.23E-02 | Up regulated in gastric carcinoma |  |
| CDX1 | -7.3 | 1.21E-03 | Up regulated in gastric carcinoma |  |
| ETV4 | -1.9 | 1.32E-02 | Up regulated in gastric carcinoma |  |
| PPP1R1B | -4.6 | 5.07E-03 | Up regulated in gastrointestinal cancers |  |
| TM4SF5 | -3.9 | 1.29E-02 | Candidate oncogene |  |
| GPC3 | -3.8 | 2.52E-04 | Up regulated in hepatocellular carcinoma |  |
| TLX1 | -3.7 | 1.79E-02 | Oncogene |  |
| PEG10 | -3.7 | 2.32E-02 | Up regulated in hepatocellular carcinoma |  |
| WNT4 | -3.4 | 2.66E-02 | Candidate oncogene |  |
| CA8 | -4.6 | 2.04E-03 | Promotes colon cancer cell growth |  |
| BCAS1 | -3.7 | 4.33E-02 | Oncogene |  |
| FAM84A | -3.4 | 2.29E-03 | Promotes colon cancer |  |
| USP2 | -2.6 | 1.19E-02 | Candidate oncogene – negative regulator of p53 |  |
|  |  |  |  |  |
| Miscellaneous | | | | |
| HHIP | -5.3 | 6.06E-03 | Inhibits Hedgehog signaling |  |
| SHISA3 | -3.9 | 1.70E-02 | Inhibits Wnt and FGF signaling |  |
| NKD2 | -3.1 | 1.02E-02 | Antagonist of Wnt signaling |  |
| LRP4 | -3.5 | 4.25E-04 | Negative regulator of Wnt signaling |  |
| DUSP8 | -1.6 | 2.51E-02 | Inhibits JNK pathway |  |
| SLC26A3 | -4.9 | 3.24E-02 | Expression inhibited by IFNG |  |
| TNFSF11 | -3.0 | 2.29E-02 | Regulator of T cells and dendritic cells |  |

**References**

1. Chu G, Qi S, Yang G, Dou K, Du J, et al. (2012) Gastrointestinal tract specific gene GDDR inhibits the progression of gastric cancer in a TFF1 dependent manner. Molecular and Cellular Biochemistry 359: 369-374.

2. Hong S-J, Oh J-H, Jung Y-C, Kim Y-H, Kim S-J, et al. (2010) DNA Methylation Patterns of Ulcer-Healing Genes Associated with the Normal Gastric Mucosa of Gastric Cancers. J Korean Med Sci 25: 405-417.

3. Katuri V, Tang Y, Marshall B, Rashid A, Jogunoori W, et al. (2005) Inactivation of ELF//TGF-[beta] signaling in human gastrointestinal cancer. Oncogene 24: 8012-8024.

4. Okugawa T, Oshima T, Chen X, Hori K, Tomita T, et al. (2012) Down-Regulation of Claudin-3 Is Associated with Proliferative Potential in Early Gastric Cancers. Digestive Diseases and Sciences 57: 1562-1567.

5. Sentani K, Oue N, Naito Y, Sakamoto N, Anami K, et al. (2012) Upregulation of HOXA10 in gastric cancer with the intestinal mucin phenotype: reduction during tumor progression and favorable prognosis. Carcinogenesis 33: 1081-1088.

6. Schofield D, West D, Anthony D, Marshal R, Sklar J (1995) Correlation of loss of heterozygosity at chromosome 9q with histological subtype in medulloblastomas. Am J Pathol 146: 472-480.

7. Bralten LBC, Gravendeel AM, Kloosterhof NK, Sacchetti A, Vrijenhoek T, et al. (2010) The CASPR2 cell adhesion molecule functions as a tumor suppressor gene in glioma. Oncogene 29: 6138-6148.

8. Yu G, Tseng G, Yu Y, Gavel T, Nelson J, et al. (2006) CSR1 Suppresses Tumor Growth and Metastasis of Prostate Cancer. Am J Pathol 168: 597-607.

9. Moniz S, Verissimo F, Matos P, Brazao R, Silva E, et al. (2007) Protein kinase WNK2 inhibits cell proliferation by negatively modulating the activation of MEK1//ERK1//2. Oncogene 26: 6071-6081.

10. Mlakar V, Stra≈æi≈°ar M, Sok M, Glavaƒç D (2009) Oligonucleotide DNA Microarray Profiling of Lung Adenocarcinoma Revealed Significant Downregulation and Deletions of Vasoactive Intestinal Peptide Receptor 1. Cancer Investigation 28: 487-494.

11. Koenig-Hoffmann K, Bonin-Debs AL, Boche I, Gawin B, Gnirke A, et al. (2005) High throughput functional genomics: Identification of novel genes with tumor suppressor phenotypes. International Journal of Cancer 113: 434-439.

12. Caretti A, Sirchia SM, Tabano S, Zulueta A, Dall'Olio F, et al. (2012) DNA methylation and histone modifications modulate the B1,3 galactosyltransferase B3Gal-T5 native promoter in cancer cells. The International Journal of Biochemistry &amp; Cell Biology 44: 84-90.

13. Tsunoda S, Smith E, De Young N, Wang X, Tian Z, et al. (2009) Methylation of CLDN6, FBN2, RBP1, RBP4, TFPI2, and TMEFF2 in esophageal squamous cell carcinoma. Oncology Reports 21: 1067-1073.

14. Taieb D, Roignot J, Andr√© Fdr, Garcia Sp, Masson Bnd, et al. (2008) ArgBP2-Dependent Signaling Regulates Pancreatic Cell Migration, Adhesion, and Tumorigenicity. Cancer Research 68: 4588-4596.

15. Hwang S, Kim K, Kim J, Kim H, Lee M, et al. (2011) Detection of HOXA9 gene methylation in tumor tissues and induced sputum samples from primary lung cancer patients. Clin Chem Lab Med 49: 699-704.

16. Dmitriev A, Kashuba V, Haraldson K, Senchenko V, Pavlova T, et al. (2012) Genetic and epigenetic analysis of non-small cell lung cancer with NotI-microarrays. Epigenetics 7: 502-513.

17. Tang Y, Shu G, Yuan X, Jing N, Song J (2011) FOXA2 functions as a suppressor of tumor metastasis by inhibition of epithelial-to-mesenchymal transition in human lung cancers. Cell Res 21: 316-326.

18. Lucas B, Grigo K, Erdmann S, Lausen J, Klein-Hitpass L, et al. (2005) HNF4[alpha] reduces proliferation of kidney cells and affects genes deregulated in renal cell carcinoma. Oncogene 24: 6418-6431.

19. Zuo H, Gandhi M, Edreira MM, Hochbaum D, Nimgaonkar VL, et al. (2010) Downregulation of Rap1GAP through Epigenetic Silencing and Loss of Heterozygosity Promotes Invasion and Progression of Thyroid Tumors. Cancer Research 70: 1389-1397.

20. Liu Q-S, Zhang J, Liu M, Dong W-G (2010) Lentiviral-mediated miRNA against liver-intestine cadherin suppresses tumor growth and invasiveness of human gastric cancer. Cancer Science 101: 1807-1812.

21. Kang JM, Lee BH, Kim N, Lee HS, Lee HE, et al. (2011) CDX1 and CDX2 Expression in Intestinal Metaplasia, Dysplasia and Gastric Cancer. J Korean Med Sci 26: 647-653.

22. Keld R, Guo B, Downey P, Cummins R, Gulmann C, et al. (2011) PEA3/ETV4-related transcription factors coupled with active ERK signalling are associated with poor prognosis in gastric adenocarcinoma. Br J Cancer 105: 124-130.

23. Vangamudi B, Zhu S, Soutto M, Belkhiri A, El-Rifai W (2011) Regulation of B-catenin by t-DARPP in upper gastrointestinal cancer cells. Mol Cancer 10: 32.

24. Lee S-A, Kim TY, Kwak TK, Kim H, Kim S, et al. (2010) Transmembrane 4 L six family member 5 (TM4SF5) enhances migration and invasion of hepatocytes for effective metastasis. Journal of Cellular Biochemistry 111: 59-66.

25. Ruan J, Liu F, Chen X, Zhao P, Su N, et al. (2011) Inhibition of glypican-3 expression via RNA interference influences the growth and invasive ability of the MHCC97-H human hepatocellular carcinoma cell line. Int J Mol Med 28: 497-503.

26. Hatano M, Roberts CWM, Minden M, Crist WM, Korsmeyer SJ (1991) Deregulation of a Homeobox Gene, HOX11, by the t(10;14) in T Cell Leukemia. Science 253: 79-82.

27. Tsuji K, Yasui K, Gen Y, Endo M, Dohi O, et al. (2010) PEG10 is a probable target for the amplification at 7q21 detected in hepatocellular carcinoma. Cancer Genetics and Cytogenetics 198: 118-125.

28. Louis I, Heinonen KM, Chagraoui J, Vainio S, Sauvageau G, et al. (2008) The Signaling Protein Wnt4 Enhances Thymopoiesis and Expands Multipotent Hematopoietic Progenitors through Œ≤-Catenin-Independent Signaling. Immunity 29: 57-67.

29. Nishikata M, Nishimori I, Taniuchi K, Takeuchi T, Minakuchi T, et al. (2007) Carbonic anhydrase-related protein VIII promotes colon cancer cell growth. Molecular Carcinogenesis 46: 208-214.

30. Collins C, Rommens JM, Kowbel D, Godfrey T, Tanner M, et al. (1998) Positional cloning of ZNF217 and NABC1: Genes amplified at 20q13.2 and overexpressed in breast carcinoma. Proceedings of the National Academy of Sciences 95: 8703-8708.

31. Kobayashi T, Masaki T, Sugiyama M, Atomi Y, Furukawa Y, et al. (2006) A gene encoding a family with sequence similarity 84, member A (FAM84A) enhanced migration of human colon cancer cells. Int J Oncol 29: 341-347.

32. Stevenson LF, Sparks A, Allende-Vega N, Xirodimas DP, Lane DP, et al. (2007) The deubiquitinating enzyme USP2a regulates the p53 pathway by targeting Mdm2. EMBO J 26: 976-986.

33. Olsen C, Hsu P-P, Glienke J, Rubanyi G, Brooks A (2004) Hedgehog-interacting protein is highly expressed in endothelial cells but down-regulated during angiogenesis and in several human tumors. BMC Cancer 4: 43.

34. Furushima K, Yamamoto A, Nagano T, Shibata M, Miyachi H, et al. (2007) Mouse homologues of Shisa antagonistic to Wnt and Fgf signalings. Developmental Biology 306: 480-492.

35. Hu T, Li C, Cao Z, Van Raay TJ, Smith JG, et al. (2010) Myristoylated Naked2 Antagonizes Wnt-B-Catenin Activity by Degrading Dishevelled-1 at the Plasma Membrane. Journal of Biological Chemistry 285: 13561-13568.

36. Li Y, Pawlik B, Elcioglu N, Aglan M, Kayserili Hl, et al. (2010) LRP4 Mutations Alter Wnt-Catenin Signaling and Cause Limb and Kidney Malformations in Cenani-Lenz Syndrome. American journal of human genetics 86: 696-706.

37. Muda M, Theodosiou A, Rodrigues N, Boschert U, Camps M, et al. (1996) The Dual Specificity Phosphatases M3/6 and MKP-3 Are Highly Selective for Inactivation of Distinct Mitogen-activated Protein Kinases. Journal of Biological Chemistry 271: 27205-27208.

38. Saksena S, Singla A, Goyal S, Katyal S, Bansal N, et al. (2010) Mechanisms of transcriptional modulation of the human anion exchanger SLC26A3 gene expression by IFN-Œ≥. American Journal of Physiology - Gastrointestinal and Liver Physiology 298: G159-G166.

39. Anderson DM, Maraskovsky E, Billingsley WL, Dougall WC, Tometsko ME, et al. (1997) A homologue of the TNF receptor and its ligand enhance T-cell growth and dendritic-cell function. Nature 390: 175-179.
